# Talking Trials: An arts‐based exploration of attitudes to clinical trials amongst minority ethnic members of the South Riverside Community of Cardiff

**DOI:** 10.1111/hex.13740

**Published:** 2023-03-02

**Authors:** Sarah Bridges, Catherine Lamont‐Robinson, Allan Herbert, Mashmooma Din, Carl Smith, Nasra Ahmed, Arafa Ali, Sudipta Bandyopadhyay, Saleema Bibi, Rossana Canu, Mariama N. G. Correia, Mamadu S. Djalo, Kense Hayan, Alka Horne, Ayesha Mita, Martina Svobodova

**Affiliations:** ^1^ Centre for Trials Research, College of Biomedical and Life Sciences Cardiff University Cardiff UK; ^2^ Social and Community Medicine, Bristol Medical School University of Bristol Bristol UK; ^3^ Cardiff University Cardiff UK; ^4^ South Riverside Community Development Centre Cardiff UK

**Keywords:** clinical trials, inclusivity, knowledge co‐production, public dialogue, public involvement, underserved communities

## Abstract

**Introduction:**

Clinical trials must include diverse participants to ensure the wide applicability of results. However, people from ethnic minorities are included in clinical trials at rates lower than expected given their share of the population. Working with South Riverside Community Development Centre (SRCDC), Talking Trials used public engagement to foster discussions around the underrepresentation of those from minority ethnic communities in clinical trials and to identify and address concerns surrounding trial participation.

**Methods:**

We conducted three workshops with 13 co‐researchers from minority ethnic backgrounds. We explored perceptions and understanding of clinical trials alongside participatory art activities to help move away from verbocentric methods of communication. These artworks formed an exhibition that was presented to the community, prompting further discussions and engagement.

**Findings:**

Co‐production workshops were an effective tool to introduce the public to trial research. With little knowledge of clinical trials at the beginning of the process, our co‐researchers formed a cohesive group, sharing initial fears and mistrust towards trials. As conversations progressed these attitudes clearly shifted. Artwork produced during the workshops was incorporated into an exhibition. Quotes and creative pieces from the group were included to reflect the themes identified. Presenting the exhibition at Riverside Festival enabled further engagement with a wider diverse community. The focus on co‐production helped build a network of individuals new to research and keen to become involved further.

**Conclusion:**

Inclusive and democratic co‐production, enriched by participatory art practices, provided a powerful means of enabling our group to create new insights and foster new relationships. Projects like Talking Trials can diversify the research process itself—for example, four co‐researchers have commenced lay research partner roles on trial management groups and a lay advisory group is in development.

**Patient or Public Contribution:**

Three members of staff at SRCDC were on the project delivery group and involved in the initial project design, subsequently helping to connect us with members of the Riverside community to work as co‐researchers. Two of the SRCDC staff are co‐authors of this manuscript. The project had 13 public co‐researchers guiding the direction of this research and creating the artwork displayed in the art exhibition.

## 1 INTRODUCTION

Clinical trials are the primary method for researchers to find out if a new drug or an intervention is safe and effective. However, if the trial population differs from the population that will actually receive the intervention (the ‘target population’), then trial results may not apply to the target population.^1^ Trial populations should, therefore, closely reflect the target population. In a diverse country such as the United Kingdom, this usually means that populations recruited into trials need to include diverse participants of different ages, sexes, races and ethnicities to ensure wide applicability. The United Kingdom has become more ethnically diverse with 15.2% of England and Wales's population identifying their ethnicity as other than White in 2019, increasing by 1.2 percentage points since the 2011 Census.^2^ In Scotland, the population identifying as minority ethnic doubled to 4% between 2001 and 2011,^3^ and in Northern Ireland, the population identifying as non‐White increased by 1.6%–3.4% between 2011 and 2021.^4^

However, existing data show people from ethnic minorities are included in clinical trials at rates lower than we would expect given their share of the population.^5^, ^6^, ^7^, ^8^

This lack of representation has both scientific and ethical implications. Failing to include a broad range of participants in trials means that results might not be generalizable to a broad population, and interethnic differences in the metabolism of different medicines will not be picked up until the drugs are being widely prescribed.^9^ Furthermore, environmental and cultural factors (such as income, education or religion) are also involved in determining response to medicines in different racial and ethnic groups.^10^ To not consider these factors during the trial design stage may result in a lack of engagement from some underrepresented groups.

Research also shows that minority ethnic patients have worse experiences when accessing treatment, and poorer survival in general,^11^, ^12^ which is evident in nearly every dimension of care. These patients face health inequalities and poor outcomes compared to the White population, with linguistic (language use/ability) and social‐cultural (mistrust towards research/health professionals, unfair access) barriers being the most commonly identified.^13^ The COVID‐19 pandemic has further amplified this issue, with people from minority ethnic backgrounds being more likely to develop serious complications from the disease.^14^, ^15^ Some trials may, therefore, require more representation of specific populations due to higher disease burden.

There is increasing interest in the need for more inclusive practice with respect to historically marginally represented groups,^16^, ^17^, ^18^ as viewpoints of individuals from outside the research community have been neglected when it comes to designing studies and reporting results.^19^, ^20^ Building long‐term relationships with underserved groups have been identified as one of the key strategies to improve the representation of underserved groups in clinical trials.^21^ This includes diversifying the research process itself by representing underserved groups within research teams (including trial overseeing committees), funding bodies and research ethics committees.

Talking Trials public engagement proof‐of‐concept project took place in the spring and summer of 2021. The project consisted of a series of conversations and workshops held with a group of co‐researchers from different minority ethnic communities centred around the Riverside area of Cardiff. Working in close partnership with South Riverside Community Development Centre (SRCDC), a local organisation with expertise in engaging with ethnic minority groups, the aim of Talking Trials was to engage in discussions surrounding the underrepresentation of those from minority ethnic communities in clinical trials, exploring views and addressing concerns surrounding trial participation, while identifying whether a co‐production approach would lead to a change in attitudes. To ensure accessibility to the Riverside community and to seek to hold our group's interest, we engaged the expertise of an artist–educator to work with the group to coproduce artwork reflecting group discussions and attitudes.

In this paper, we describe conversations held with our co‐researchers, along with changes in attitudes towards clinical trials that were seen across the course of these workshops. We discuss the participatory art methods used to draw out attitudes and perspectives on involvement in clinical trials.

## 2 METHODS

**Figure 1:**
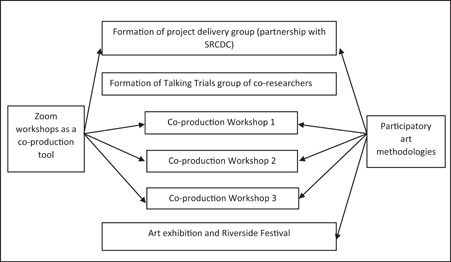
Timeline of activities. SRCDC, South Riverside Community Development Centre.

The timeline of activities undertaken in Talking Trials is shown in Figure 1. Each of these is described below.

### 2.1 Formation of project delivery group

Guidance responding to the UK National Institute for Health Research include project on how inclusivity in trials should be addressed with respect to ethnic minorities suggests that a key strategy should be the building of trusting partnerships with community organisations that work with ethnic minority groups.^22^ Close partnership with SRCDC was instrumental in forming a group of co‐researchers from minority ethnic backgrounds. A project delivery group was formed, consisting of two principal investigators from the Centre for Trials Research (CTR) (one British, one Czech), an artist (Irish) with a strong background in participatory arts methodologies and three SRCDC representatives (one of Pakistani, one of Sudanese and one of Welsh origin). The group planned the delivery of and informed and influenced the content of the workshop series, advising on recruitment strategies for a group of co‐researchers from minority ethnic communities.

The initial project delivery group meetings discussed initial ideas as to why people from minority ethnic backgrounds might be less likely to take part in trials, key factors that might decision making and what recruitment strategies could be implemented to increase the likelihood of people from these backgrounds taking part in clinical trials. This meeting was also used by the artist to pilot her prospective participatory arts approaches.

### 2.2 Formation of the group of co‐researchers

Thirteen residents of Riverside and surrounding areas of Cardiff were recruited as co‐researchers to participate in discussions aiming to develop new insights into issues of the underrepresentation of those from minority ethnic communities in clinical trials.

South Riverside, the location of SRCDC, was identified as a suitable location for the project as this area has a large minority ethnic population (51% compared to just 15% of the Cardiff population as a whole), and the largest Asian population in Cardiff (35.5%).^2^, ^23^

Co‐production is an approach in which researchers and the public work together, sharing power and responsibility from the start to the end of the project, including the generation of knowledge. The focus on co‐production was a deliberate attempt to foster a network of individuals new to research, thereby democratising research; we wanted new voices to shift the ways in which we work. It was our aim from the outset for co‐researchers to dictate the direction of our workshops. We were keen to ensure that our conversations formed a positive experience for co‐researchers, breaking down barriers between researchers and the public, and forming an intimate and close‐knit group within which co‐researchers felt safe to give their views and produce new knowledge. Presenting the process as a journey for all of us ensured a two‐way flow of cultural knowledge.

We strongly felt, given the four key principles of co‐production (namely equality, diversity, accessibility and reciprocity),^24^ that some compensation should be offered to the group to put them on an equal footing with the principal investigators and demonstrate decolonisation of research; for the CTR researchers to be paid for input into the group but for the co‐researchers to not be would have created an inequality from the outset. As this project was funded as a public engagement activity, financial incentives to participants were not deemed an eligible cost. However, with a clear steer from the community members that incentivisation would be important to reflect their participation in the group, we secured permission from the funder to provide £20 supermarket vouchers for each workshop.

### 2.3 Co‐production workshops 1, 2 and 3

A group of 13 co‐researchers, 3 members of the project delivery group, 2 principal investigators and the project artist participated in three workshops that took place over a period of 3 months. At the first workshop, a creative icebreaker helped ease co‐researchers into discussing identity and background. The responses to the icebreaker had a strong focus on bodily senses—seeing colours, touching sand, hearing birdsong, smelling flowers—which led to discussions about the body, health and medicine, with the group finding common threads through their lived experiences regardless of background. This helped cement the group as a cohesive unit from the outset.

At each workshop, we introduced a certain aspect of the clinical trials landscape, focussing on the importance of trials reflecting a diverse population to ensure that trial results are applicable to the whole population rather than just those who traditionally tend to participate in trials. We explored levels of awareness amongst the co‐researchers, along with prior perceptions of trials and medical research in general. The group looked at the importance of inclusivity and explored ways that the public can become involved in research (e.g., by becoming a research partner in a clinical trial management group).

Examples of participant information sheets from the CTR (including some using infographics) were reviewed by the group to identify points that may prevent those from minority ethnic backgrounds from entering trials. It became clear though, with the co‐researchers directing the research themselves, that experiences of COVID‐19 would prove integral to discussions, so while the focus remained on clinical trials, in‐depth discussion of patient information materials was shelved as the group discussions were taken in a different direction.

### 2.4 Participatory art methodologies

Participatory practices are characterised by interpersonal complexity, and emergent innovations in arts‐based research offer the potential to generate and hold multifaceted data.^25^ Researchers often directly engage with the selected methodologies themselves, demonstrating their commitment to authentically developing joint knowledge through diverse media with a breadth of cultural relevance.^26^ These methodologies are increasingly utilised in participatory research to legitimise diverse knowledge and languages through creative enquiries. Informal arts engagements provided opportunities to complement quantitative and verbocentric aspects of this research, gathering rich, holistic data and perspectives which may have otherwise escaped representation.

The creative element of our workshops was facilitated by the project artist, to generate nonverbal data and to respond to emerging individual interests with diverse media/materials. A package of art materials was sent to co‐researchers to work with throughout the three workshops and beyond. The group sessions supported multisensory pathways while the co‐researchers processed research materials and related topics of discussion. All workshop participants (co‐researchers but also the group facilitators) selected and handled ‘art’ materials that connected with their feelings and thoughts about the topics under discussion. Each group then shared tactual/visual metaphors representing their unique enquiries and triggering other knowledge.^27^

### 2.5 Zoom workshops as a tool for co‐production

The COVID‐19 pandemic disrupted face‐to‐face research projects worldwide, with restrictions on travel and social contact introduced to avoid further health risks. As a solution to the new landscape where attending in‐person meetings became prohibitive, we held the workshop series over Zoom in the Spring and Summer of 2021.

### 2.6 Art exhibition and Riverside Festival

Art produced by the co‐researchers formed the basis of a series of eight panels curated and designed by the project artist, featuring contributions from every member of the group. We utilised this exhibition for further wider community engagement at the Riverside Festival in summer of 2021, where these panels provided a forum for further discussions about clinical trials with other members of the Riverside community. We also provided a space for children and their families to make use of similar materials to those that had been used in the Talking Trials workshops, allowing them to create their own pieces of art.

### 2.7 Feedback surveys

To supplement the qualitative data generated by the creative arts approach, co‐researchers were asked to complete a baseline survey at the beginning of the process and then again after the workshops had been held. These surveys were intended to capture whether these co‐production workshops could lead to a change in attitude towards clinical trials as the project progressed, along with documenting whether co‐researchers became more or less likely to attend other science or university events.

## 3 RESULTS

### 3.1 Recruitment

Recruitment to the group of co‐researchers proved to be a major challenge. Although flyers advertising the group were delivered to every house in the Riverside ward of Cardiff, this approach did not prove effective. As discussed, community members fed back the incentivisation would be vital, so funds were reprofiled to allow the payment of supermarket vouchers to the co‐researchers. The SRCDC project delivery group members then worked with various local community groups to advertise the project and recruit members. These already existing links proved vital in accessing community members to form our co‐researcher group.

The ethnicity make‐up of the co‐researcher group was as follows: Indian (*n* = 3), Pakistani (*n* = 1), Somali (*n* = 2), Bissau‐Guinean (*n* = 4), Sudanese (*n* = 2) and Italian (*n* = 1). The gender was split between 11 females and 2 males as recruiting male participants was particularly challenging (this mirrors the fact that our partner community organisation's service users are also predominantly female). The age distribution of the group (limited to the 11 co‐researchers who returned this information) was as follows: 16–24 (*n* = 1); 30–34 (*n* = 2); 35–39 (*n* = 1); 40–44 (*n* = 2); 45–49 (*n* = 1); 55–59 (*n* = 3); 60–64 (*n* = 1).

### 3.2 Main themes

**Figure 2:**
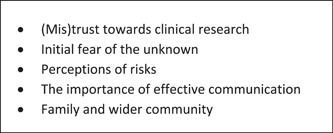
Main themes.

Figure 2 provides a summary of the main themes identified during our workshop discussions. Each of these themes is then further discussed below.

### 3.3 (Mis)trust towards clinical research

There has been considerable research into the barriers associated with minority underrepresentation in research and there is consensus that participant trust in research and research institutions is one of the most important factors. Our conversations confirmed this as the word ʻtrust’ was mentioned repeatedly. This was often extended to mistrust towards the ‘majority’ society and its institutions (i.e., the government or the police), where the co‐researchers felt bonds of trust had been repeatedly broken.

Co‐researchers also mentioned the spread of misinformation amongst their communities specifically related to the development of COVID‐19 vaccines and the belief that the vaccine would result in disabilities as well as misconceptions that there are two different kinds of vaccine: one for the majority British population, and one for minority communities.

### 3.4 The initial fear of the unknown

At the beginning of the first workshop, and without providing any introductory information, we asked the group an open question, ‘what comes to mind when we say clinical trials’. The word ʻscary’ was used frequently, with metaphors used to express this fear including white laboratory coats, impersonal hospital beds, syringes, and patients dying. While the altruistic desire to help others by taking part in research was mentioned by co‐researchers, this was not as strongly expressed as the fear of things going wrong.

As we progressed through our workshops the initial fear diminished. At our third meeting, the group reflected on how these workshops had allayed some of their fears and there was consensus that clinical trials now inspired positive feelings. The group stated that they had started noticing information about clinical trials more than they had previously, paying attention to items on the news. The whole group stated that they would be interested in future workshops on similar topics.

### 3.5 Perceptions of risks

Perceptions of risk and concern about serious and long‐term side effects played a key role in the co‐researchers' discussions. Reaching a decision to take part in a clinical trial would involve weighing multiple factors for and against, with the group emphasising the importance of receiving all available information. Important factors to consider would include information on the length of time they would be in the trial, identifying a point of contact to discuss any concerns and side effects, travel requirements, impact of their participation on their existing commitments (employment and childcare) and how many other participants were involved in the trial and whether they could connect with others to talk about their experiences.

### 3.6 The importance of effective communication

Added to the lack of trust in medical research, in general, the group also discussed the more interpersonal aspects of a potential participant's relationship with a researcher. The level of trust and confidence in this person was viewed as playing more important a role in decision making than the patient information sheet. The group felt that they were more likely to trust information about the trial if it was explained to them by someone they trust, emphasising the significance of this confidence as a key factor in the decision‐making process.

While trust in the messenger/researcher explaining the trial was paramount, the accessibility of the written information given to potential patients was also important. Examples of patient‐facing information provided to the group were viewed as too lengthy and difficult to comprehend, even more so for those with English as a second language. Some of the group found the information intrusive and alienating (specifically information related to pregnancy and how this would affect trial participation). The provision of participant information in community languages was viewed as a contributory factor that would help build trust.

### 3.7 Family and the wider community

Family and wider community views were discussed as factors contributing to the decision process. The group considered the concept of stigma and reflected that participating in a trial would be stigmatising or shameful in some communities.

Responses given were informed by the group's own life experiences, with anecdotes about their own health and encounters with doctors and those of close family members. The group's response to clinical trials is so interwoven with lived experience that these needed to be discussed and reflected upon.

### 3.8 The creative process and Riverside Festival 2021

Under the facilitation of the project artist, we explored perceptions and understandings of clinical trials alongside engaging in participatory art activities. Apart from fostering group learning, this process proved to be instrumental in overcoming the language barriers of some of our co‐researchers.

Production of artwork throughout the workshops was valued as an integral part of the project. The organic process of meaning‐making and sharing, both in dialogue and through materials, led to increased confidence in exploring visual and tactual ways to represent ideas. Several of the co‐researchers continued to develop their creative enquiries at home. Common overarching themes discussed throughout the workshops were later identified and shared back for further discussion. The artist collaborated with the co‐researchers in the representation of their artworks in relation to the distilled themes, and this was incorporated into an exhibition (see Supporting Information: Supplementary File 1).

Approximately 200 people passed through the Talking Trials stand at Riverside Festival, with some of the co‐researchers also participating in guiding visitors through the exhibition and the artwork they had created.

The exhibition proved popular with the festival attendees who were able to see and touch the co‐researchers’ textiles and artworks. Common themes discussed with members of the public included remuneration for trial participation leading to discussions surrounding the differences between academic and commercial clinical trials, and reflections on peoples’ own medical history: cancer diagnoses, lifesaving medicines and procedures and where they would be without these. The responses we had when talking to the public were overwhelmingly positive, with appreciation expressed for the work of researchers and medical research in general.

### 3.9 Co‐researchers’ feedback surveys

One co‐researcher noted in the baseline survey that they (or their close family/friends) had previously participated in a clinical trial. Six co‐researchers said they were very interested in science, and three said they were somewhat interested. None of the group expressed a disinterest in science, so the group may have been self‐selecting for this reason, as those disinterested may not have put themselves forward to participate. Three of the group had a qualification in a scientific field and one was working towards a qualification at the time of the survey.

When asked about participating in a clinical trial, the responses included worries about potential side effects and the need to obtain information about the trial before deciding. Two of the nine respondents said they would take part in a trial provided the research topic was in their interest, and that this would make them feel more in control by taking a more active role in their family's healthcare. When asked about how best they would like to be presented with information about trials before deciding to participate, most of the group preferred mixed methods of presenting the information, with a combination of written, digital and illustrated information.

When asked whether they would like to have the chance to participate in designing clinical trials, the majority said they would, if the topic of the trial was of interest and the involvement fitted in with their family and work commitments. Taking part in research was also viewed as a way of representing the voice of their communities.

Seven responses were received from the follow‐up survey sent out 4 months after the completion of the project. All seven respondents stated that they had discussed Talking Trials with their friends and family, and all stated that they knew more about clinical trials than they had at the start of the project. Five respondents stated that they were either slightly or much more interested in science now than they had been at the start, and the other two said their level of interest in science was the same as it had been at the beginning.

The participatory art element was the respondents’ favourite part of the process. Six respondents said they would be more likely to take part in a trial following their participation in Talking Trials. When asked again how they would prefer participant information to be presented, the respondents were split between purely written and purely pictures and diagrams. None of them preferred purely digital means of receiving patient information. They referred to the need to adjust the scientific language used when presenting information to potential participants who do not speak English as their first language.

When asked if they would like a chance to take part in designing clinical trials so they are more accessible, most of the responses were positive provided the topic of research was of interest and would make a beneficial impact on people's lives. The responses also mentioned the need for more awareness (with further projects like Talking Trials) and for using less academic language. One respondent answered no and explained that their negative response related to their worries about lacking the expertise to give considered opinions.

### 3.10 Future opportunities for co‐researchers

Eight co‐researchers have expressed an interest in continuing to work with CTR and different options are being explored to continue this group in an advisory capacity. Public involvement in research training has been provided to them in collaboration with Health and Care Research Wales, and mentoring will be provided to those who wish to become research partners or form an advisory group for lay review of clinical trial design at CTR. Four of the group have so far gone on to become CTR research partners.

## 4 DISCUSSION

### 4.1 Summary of results

Talking Trials was intended to combine co‐production workshops with textual and visual arts‐based methodologies to explore meaningful and active involvement of people from minority ethnic backgrounds in clinical trials, allowing us to move beyond language barriers. This approach used within the context of a clinical trials unit was unique and challenged existing practices around trial research (e.g., issues surrounding lack of recruitment, communication and widening participation).

The value that the participatory arts elements added to the project cannot be underestimated—the group all reported that these aspects were their favourite part of the workshops, with working together to create the art exhibition cementing the cohesion of the group. It is doubtful that simple focus groups working through written materials would have achieved the same levels of intimacy.

Using Zoom workshops with co‐researchers as a method of co‐production in the research process has been shown to be a feasible and acceptable method. Given the backdrop of the COVID‐19 pandemic, all our co‐researchers were already using Zoom routinely in everyday life (Riverside was identified as an area of need and its residents were amongst the recipients of the government laptop scheme to facilitate remote education by this time). The co‐researchers settled into a close and cohesive group, freely sharing their fears, worries and previous life experiences, and as the conversations progressed, their hopes and enthusiasm for becoming involved further.

Moving the workshops to the virtual realm had unexpected benefits with the workshops fitting well around co‐researchers’ work and home lives. Virtual meetings provided an intimacy that may have been missing from in‐person meetings, with family members and children dropping into view to say hello, and people going about their everyday lives as we spoke.

Awareness of clinical trials at the start of this process was relatively low, with discussions around fears, mistrust, and perceptions of people in white coats coming towards them with syringes. The change in attitude through the course of these three workshops was very apparent, with warmer attitudes and much less apprehension about clinical trials as time went on. The key turning point was the realisation that all currently approved drugs and medications used by the NHS have been through the clinical trial process, and access to medications like paracetamol or asthma medication is due to other people's previous participation in clinical trials. This point made many of the group reflect on their previous attitudes, and switch from a negative to a positive view of clinical trials.

We wanted participation in this group to be of benefit to the co‐researchers. They fed back that discussing fears and hopes with other members of their local community, along with creating artwork that went on to form an exhibition, were very positive experiences. Several of the group informed us that they had been discussing their Talking Trials experiences in their own communities, providing a conduit between group discussions in the workshops and their friends and family.

### 4.2 Comparison with other evidence

There is increasing recognition of the need for more inclusive practice with respect to historically marginally represented groups,^16^, ^17^ particularly as viewpoints of individuals from outside the research community when designing studies and reporting results have long been neglected.^19^, ^20^, ^28^ However, this development is yet to be translated through to clinical trial practice.

The Talking Trials project used qualitative methods to examine attitudes to participation in clinical trials. Despite methodological discussions about how to include the research participant's voice within clinical trials, particularly in relation to the recognition of the importance of qualitative data, this has not typically moved beyond using qualitative methods as a means to help explain and interpret quantitative results. There is a real need for need for more participatory and inclusive methods to widen access to minority ethnic communities and for trialists to ʻlook beyond focus groups and interviews’^29^ to expand the extremely limited diversity of qualitative methods currently being used.^30^

Art‐based methods have the potential to enhance the engagement of both participants and audiences, make research accessible beyond academia and generate data complementary to interview‐based methods.^31^ Over the last decade, creative interventions within the research have highlighted how scientific/artistic collaborations can lead to systemic change in clinical practices.^32^ Moreover, participatory arts methodologies have proven effective in both robustly addressing barriers to healthcare and transcending differences across historically marginalised communities.^33^

In terms of current clinical trials community engagement, several initiatives explore digital arts enquiries including highly relatable, visual story‐telling aimed to boost trial diversity,^34^ rigorously co‐developed visual narratives and materials supporting health literacy^35^ and an in‐depth exploration of the inclusion challenges, strengths, weaknesses and practical applications of digital technologies.^36^

Talking Trials closely aligns with the above projects in ethos and aspirations. Despite being online interventions, holistic multisensory engagement in the arts sat at the core of these discursive, exploratory workshops. Visual, spoken and text documentation suggested increasing confidence in research literacy, critical enquiry and artistic skills plus a growing sense of agency in terms of self‐advocacy.

The move to online workshops in the time of the COVID‐19 pandemic has been acknowledged as a useful method to generate rich data as well as benefitting both researchers and participants in democratising research processes.^37^ Other benefits such as geographical flexibility and saving time for participants have been identified and might in some cases offer more practical means for the patient and public involvement in comparison to an in‐person communication.^38^

### 4.3 Limitations

The Talking Trials group was a small gathering of people from a defined geographical area in Cardiff. We used the benefits of an intimate group to unweave rich stories and viewpoints from members of that group that may not otherwise have been forthcoming had we taken this to a larger group setting.

Focussing on the small urban area of Riverside means that our results might not be easily extrapolated to other settings (e.g., rural communities).

As this was primarily a public involvement project, we did not initially seek co‐researchers' informed consent to participate in the project evaluation. This would have allowed us to make their involvement and role in the process more explicit in this paper by giving them a voice by directly quoting them.

## 5 CONCLUSIONS

The underrepresentation of those from minority ethnic communities in clinical trials brings into question the validity of these trials and the applicability of trial results to all members of society. With poorer outcomes in general reported amongst minority ethnic patients, Talking Trials has pinpointed four themes that proved key to our discussions: mistrust towards clinical research, the initial fear of the unknown, perceptions of risks and the importance of effective communication. These themes will be further explored in future work.

Since the spotlight of COVID‐19 on health inequalities has brought these issues to the fore, there have been considerable strides forward amongst clinical trial units in trying to address issues of inclusion and diversity. While internal frameworks to combat inequality are much needed, we would argue that this needs to go in parallel with leaving the trial's office and going to meet people where they live. The importance of going out and having real conversations with people was vital to the success of Talking Trials, both in demystifying clinical trials researchers and the work that we do, but also in building up relationships with people and their families that can and will be built upon as we continue to work with them in future projects.

## AUTHOR CONTRIBUTIONS

Martina Svobodova and Sarah Bridges developed the study design with feedback from all authors. Martina Svobodova and Sarah Bridges took the lead in writing the manuscript. All authors provided critical feedback and helped shape the manuscript and read and approved the final manuscript.

## CONFLICT OF INTEREST STATEMENT

The authors declare no conflict of interest.

## Supporting information

Supporting information.

## Data Availability

Data sharing is not applicable to this article as no new data were created or analysed in this study.
