# Supplementary material for: Talking Trials: An arts‐based exploration of attitudes to clinical trials amongst minority ethnic members of the South Riverside Community of Cardiff
Source: Health Expect. 2023 Mar 2;26(3):1236–45. doi: 10.1111/hex.13740 (PMC10154814; doi:10.1111/hex.13740)
Supplement: Supplementary file 1 — Supporting information. [file HEX-26--s001.pdf]

CONVERSATIONS ACROSS COMMUNITIES

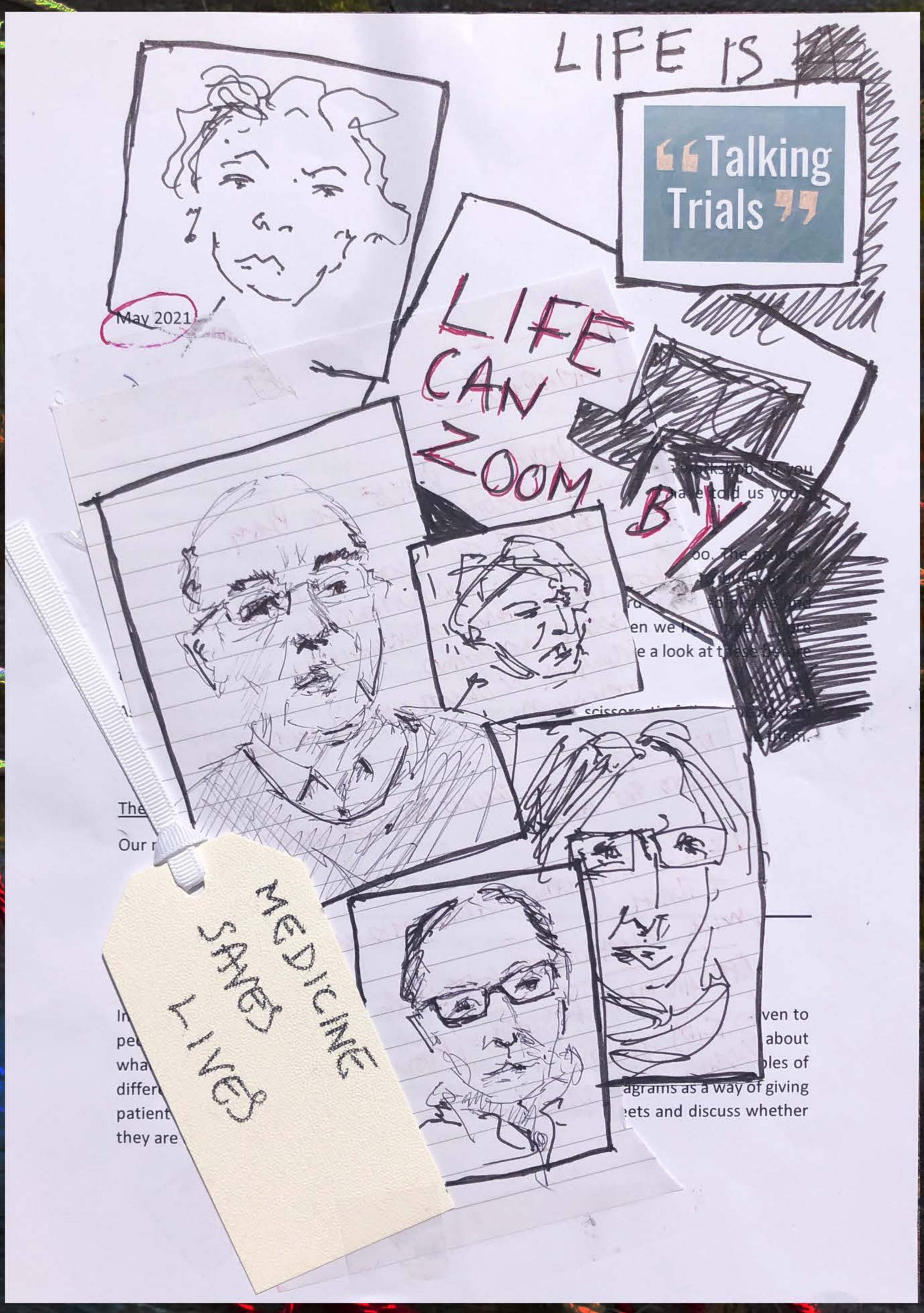

For research to improve the health of our communities, that research needs to represent the interests of everyone in them. To achieve this, we need to start talking to communities traditionally excluded from research and more importantly, start listening to what they say.

In the summer of 2021 we held a series of workshops with a group of Riverside residents from minority ethnic backgrounds. We discussed clinical trials – what they are and why they are important. We explored perceptions and views on clinical trials amongst the group, and each of us produced several items of artwork alongside these conversations. These pieces of artwork have been incorporated into an exhibition from artist-facilitator Catherine Lamont-Robinson. Quotes and pieces from the group have been included to reflect the themes identified.

Sarah Bridges and Martina Svobodova
Talking Trials Lead Researchers
Centre for Trials Research

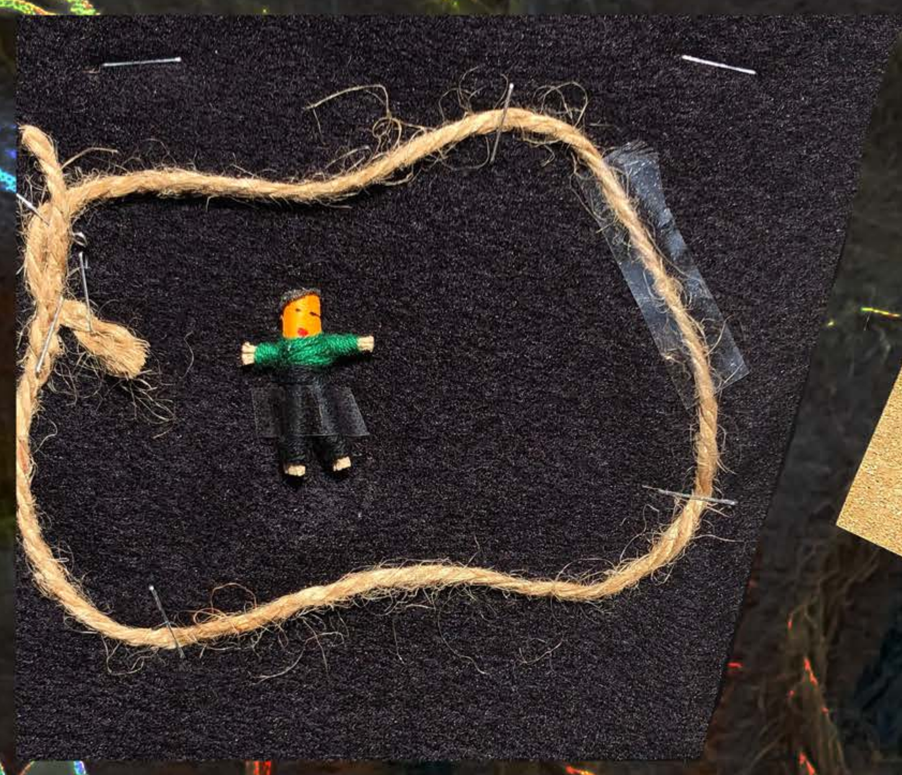

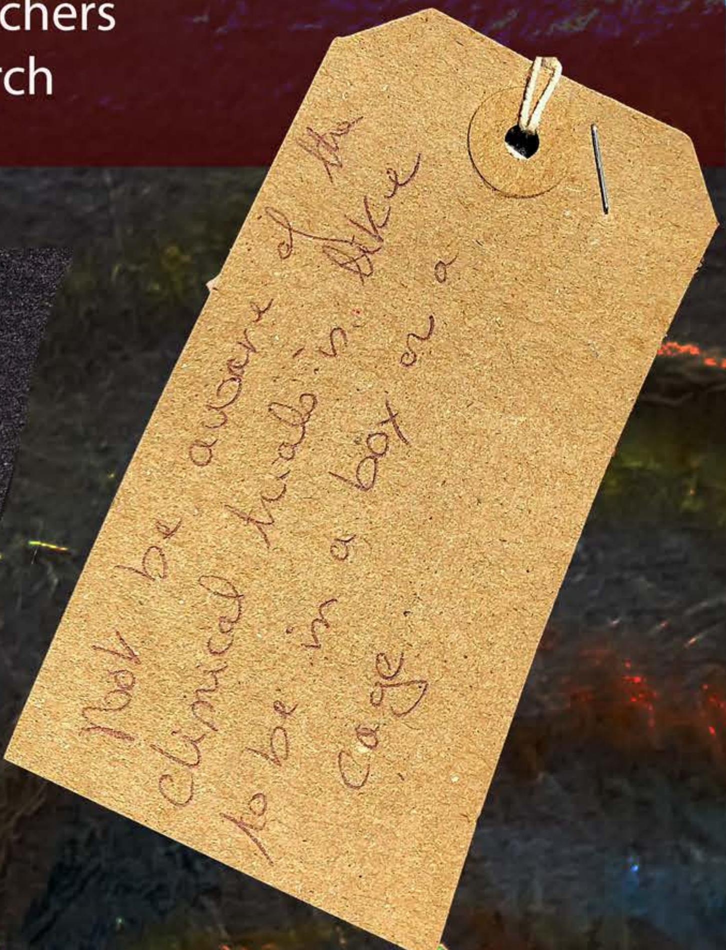

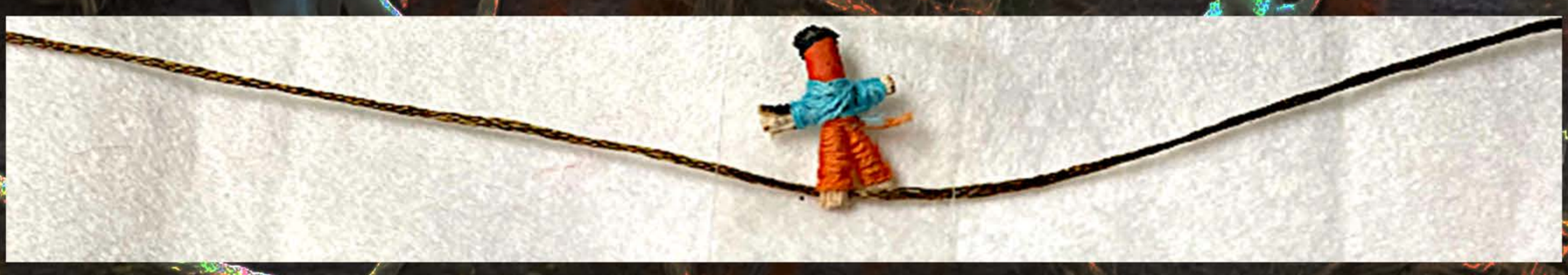

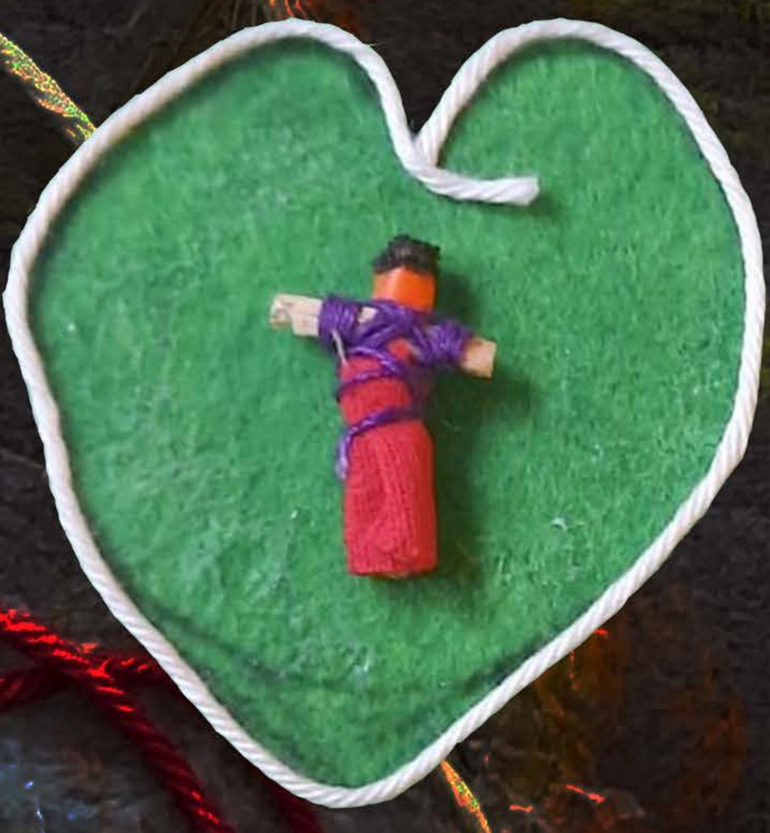

Keeping the patient at the heart of the research

WHAT IS COMMUNITY ?

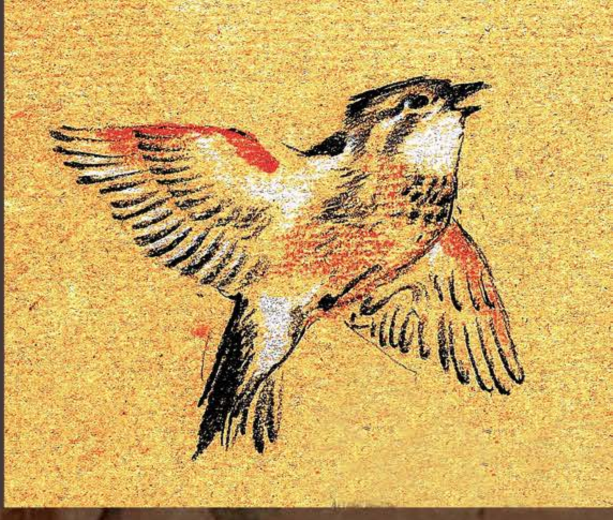

I see my family sharing and gathering a support network

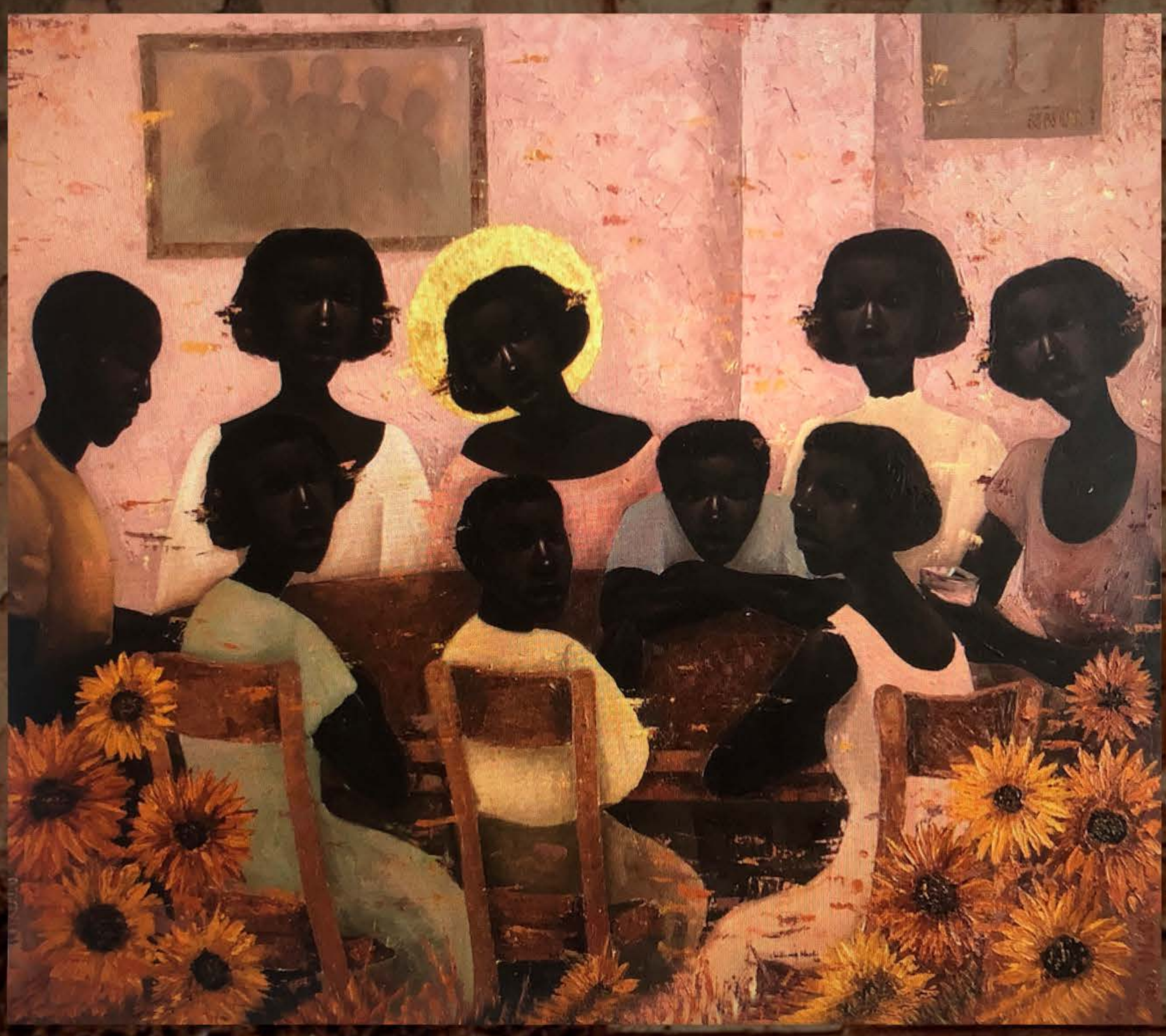

Love and genuine care and concern for others

As a community we have our own shells around us...

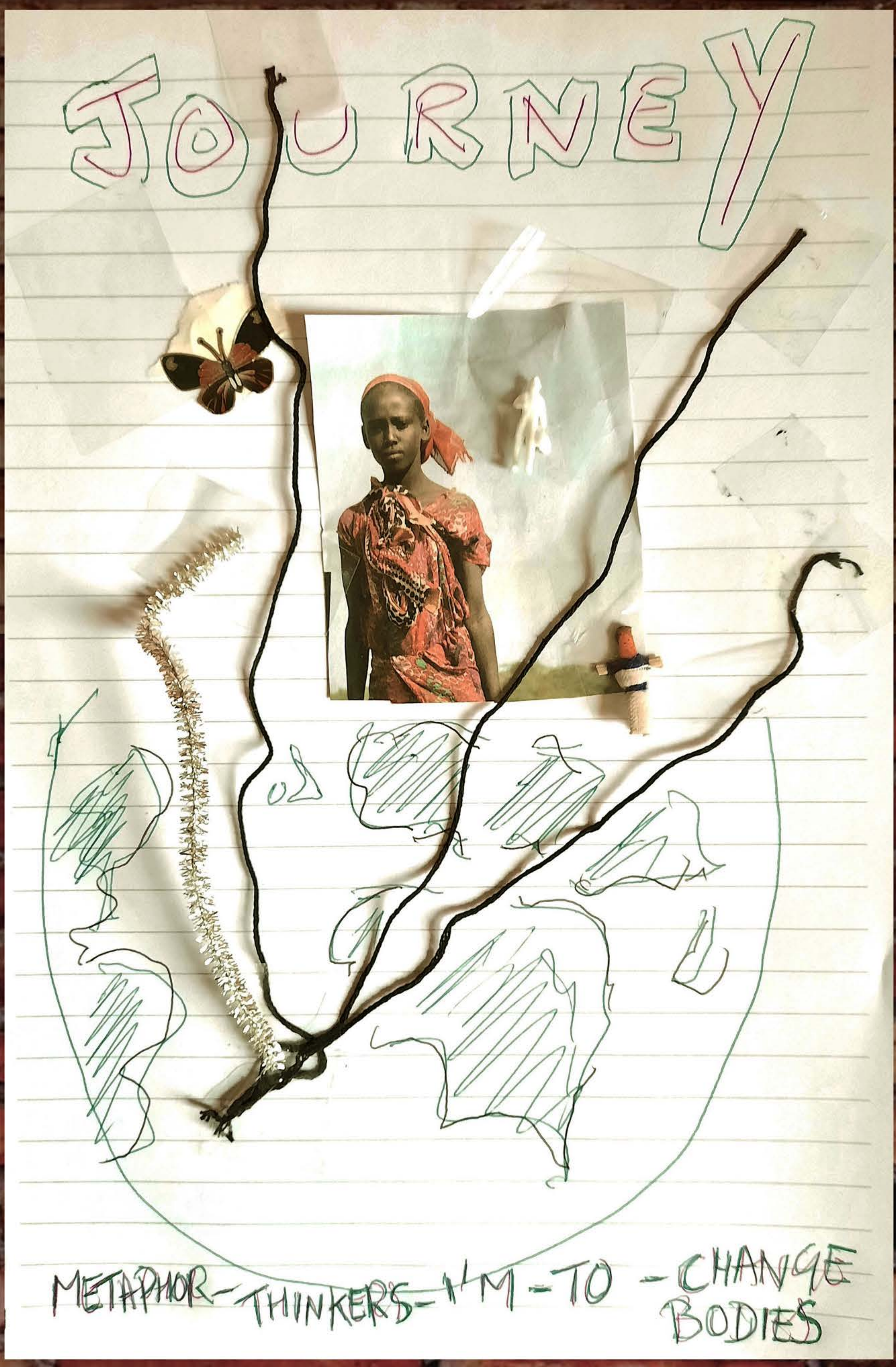

... our own mental blocks

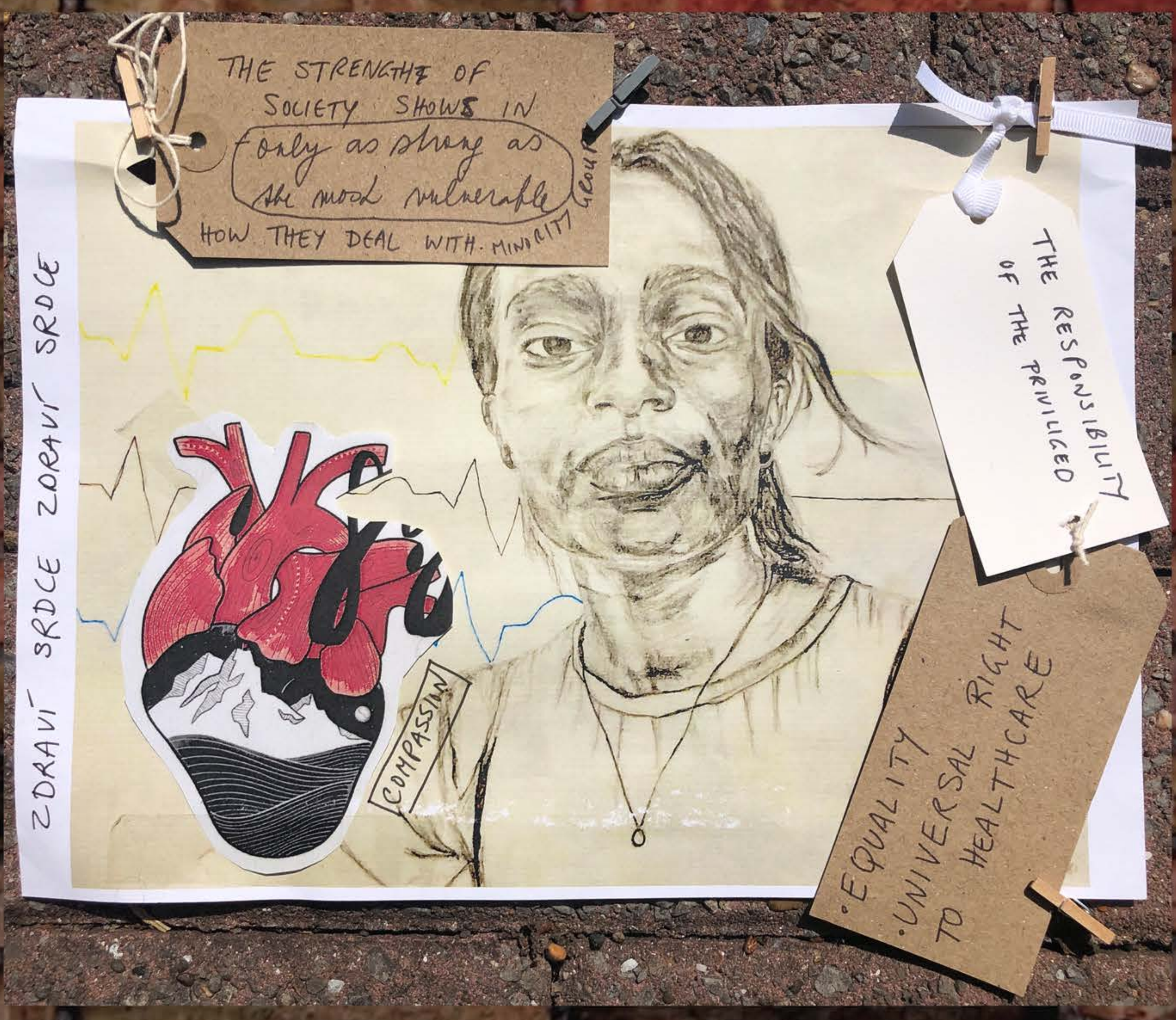

“Talking Trials”

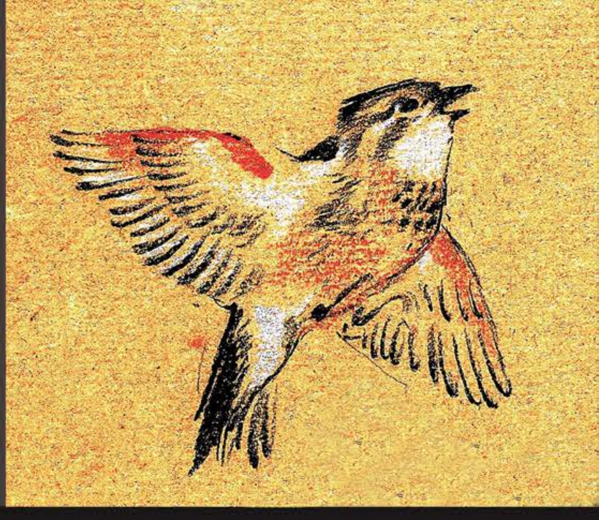

A STEP INTO CLINICAL TRIALS

Your body is a unique laboratory

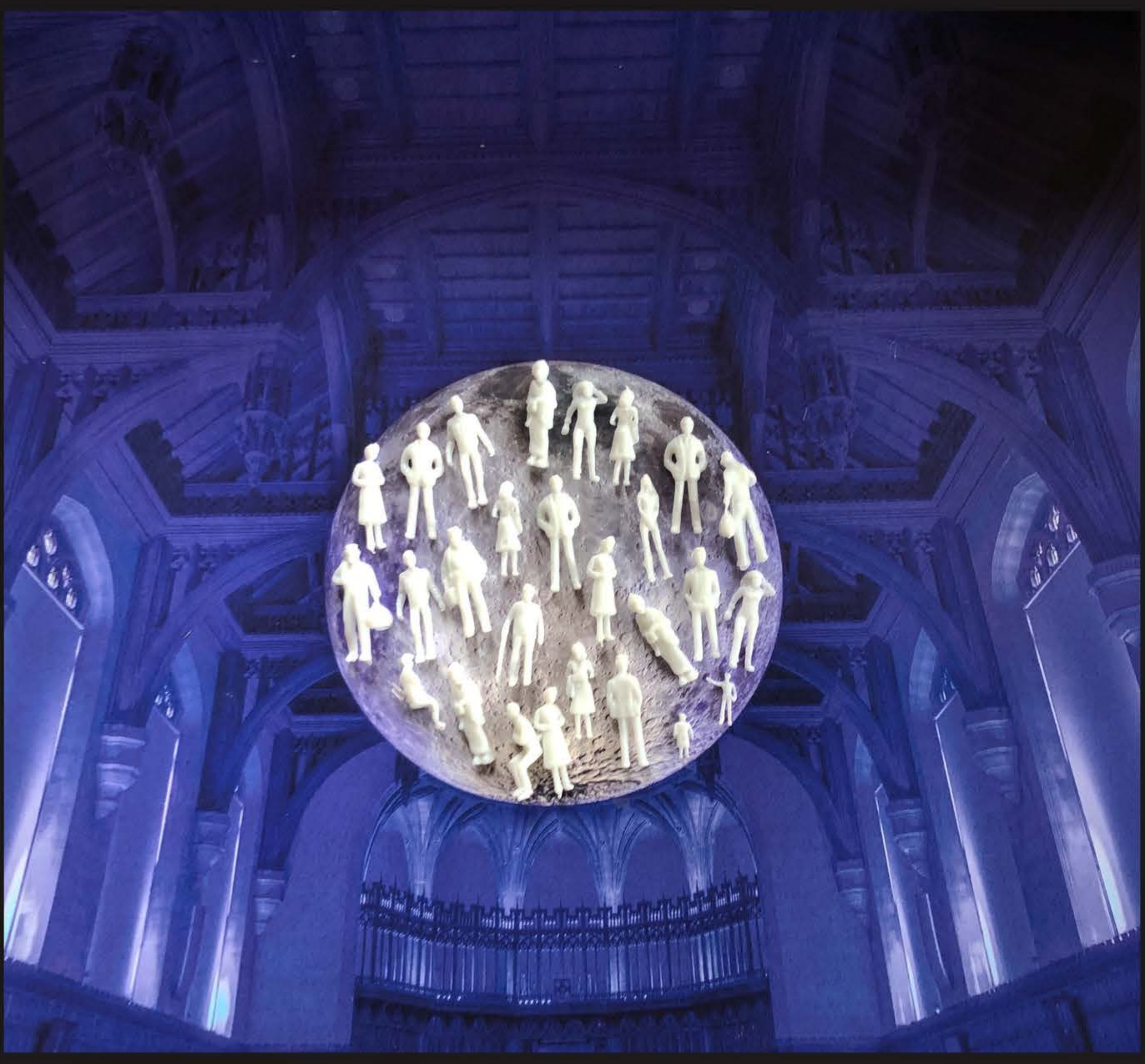

Yet all bodies are connected

All will not flow, until the hearts and souls of all are engaged on the journey of the clinical trial

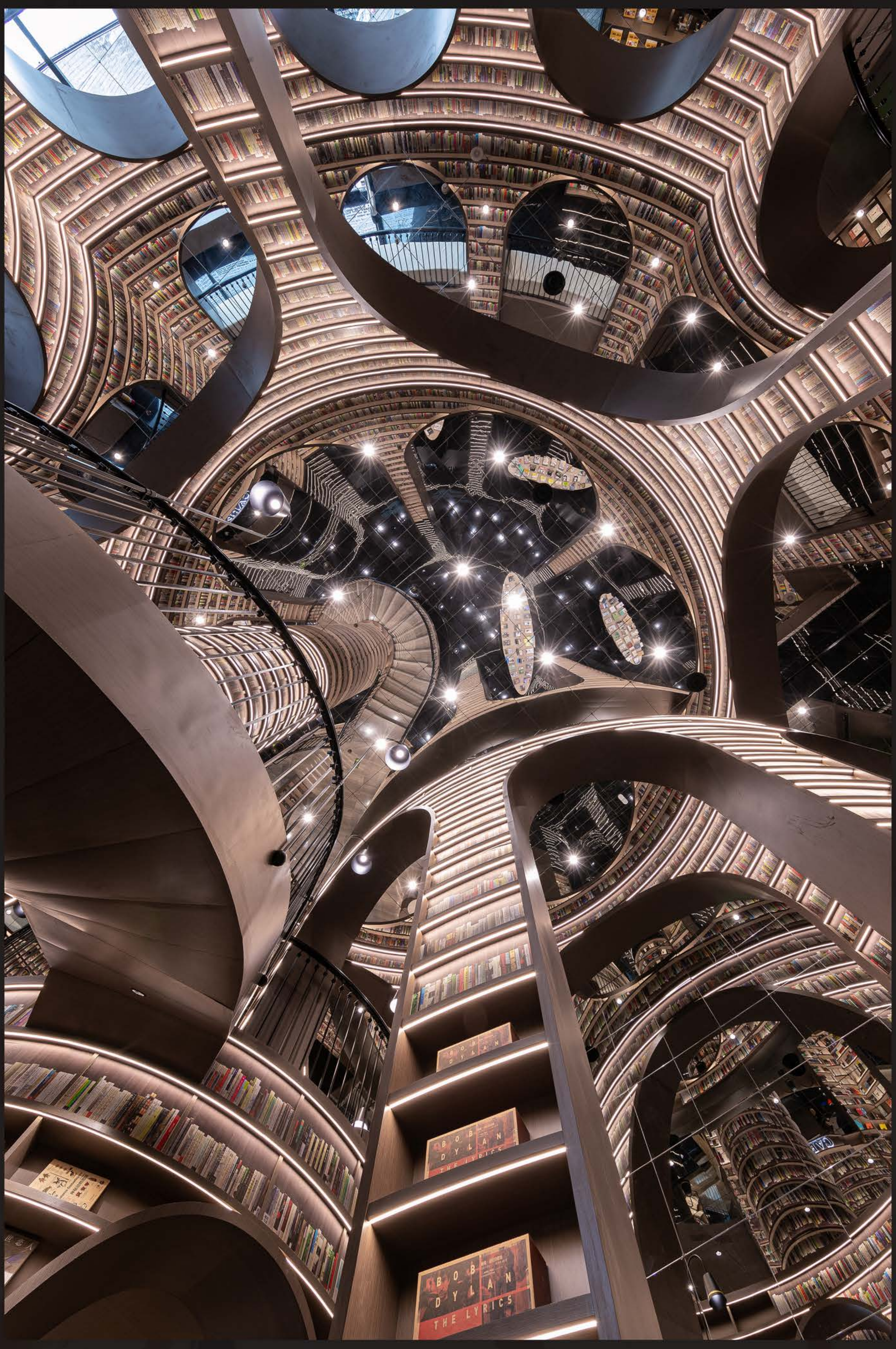

Brave to enter the unknown

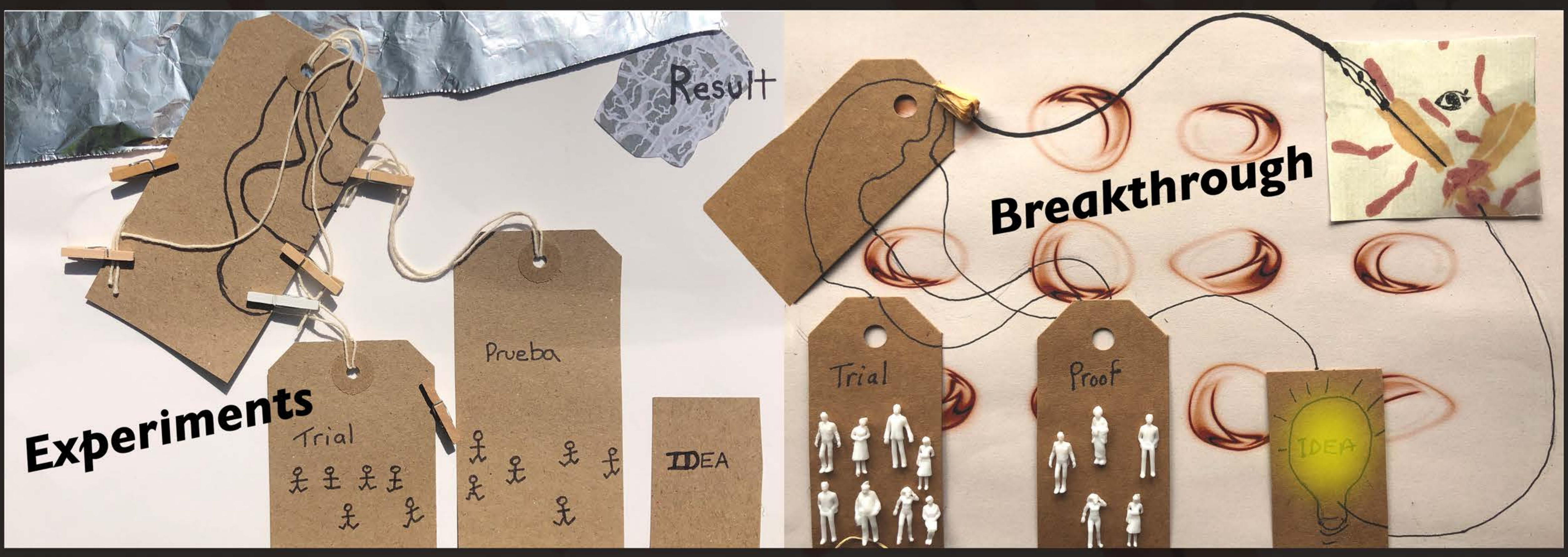

Talking Trials

FEARS AND HOPES

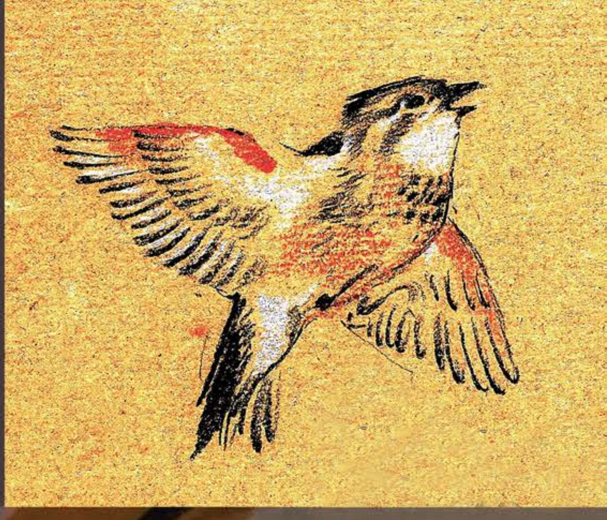

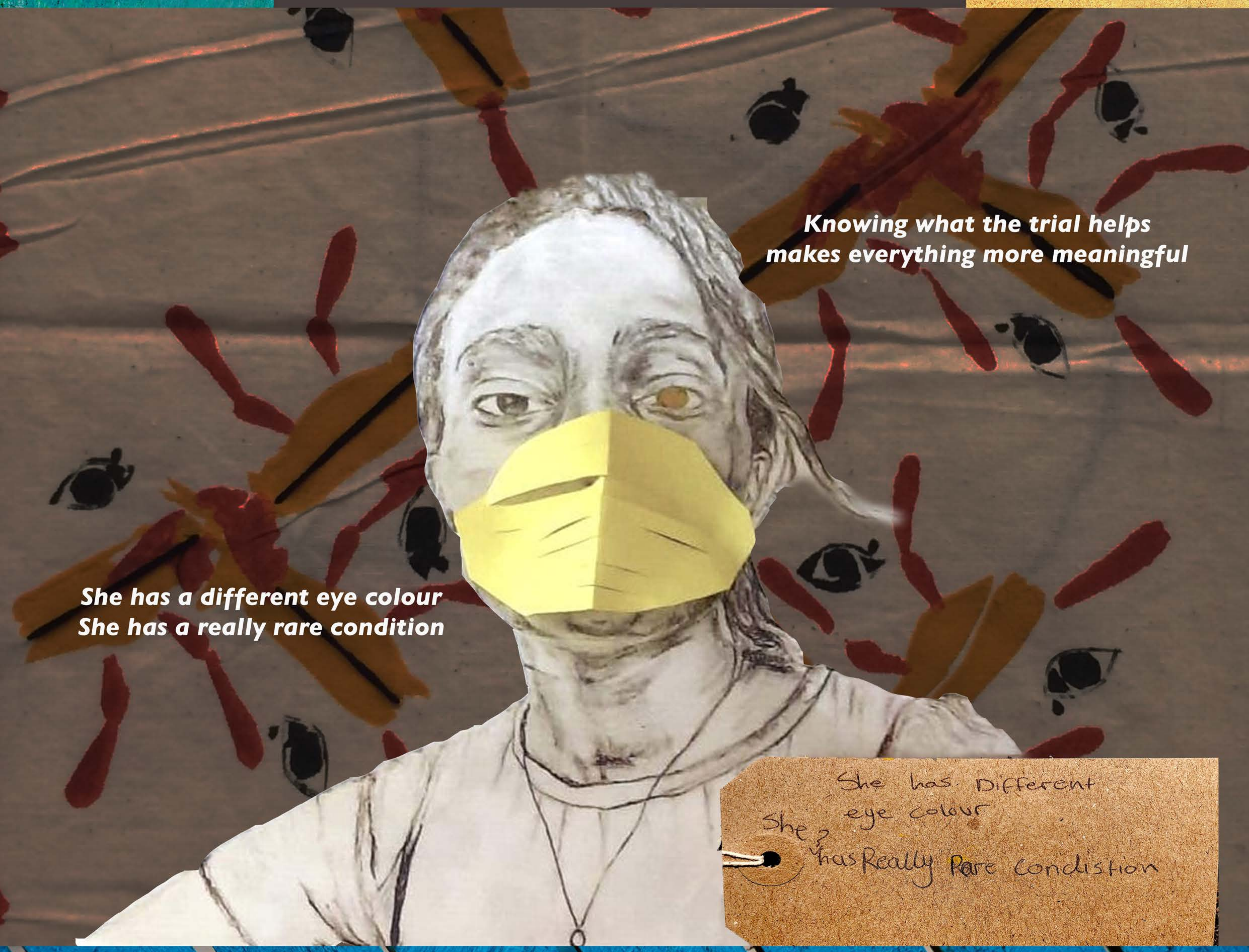

Knowing what the trial helps makes everything more meaningful

She has a different eye colour
She has a really rare condition

We need lots of detail, the ins and outs - pros and cons - we have to understand EVERYTHING

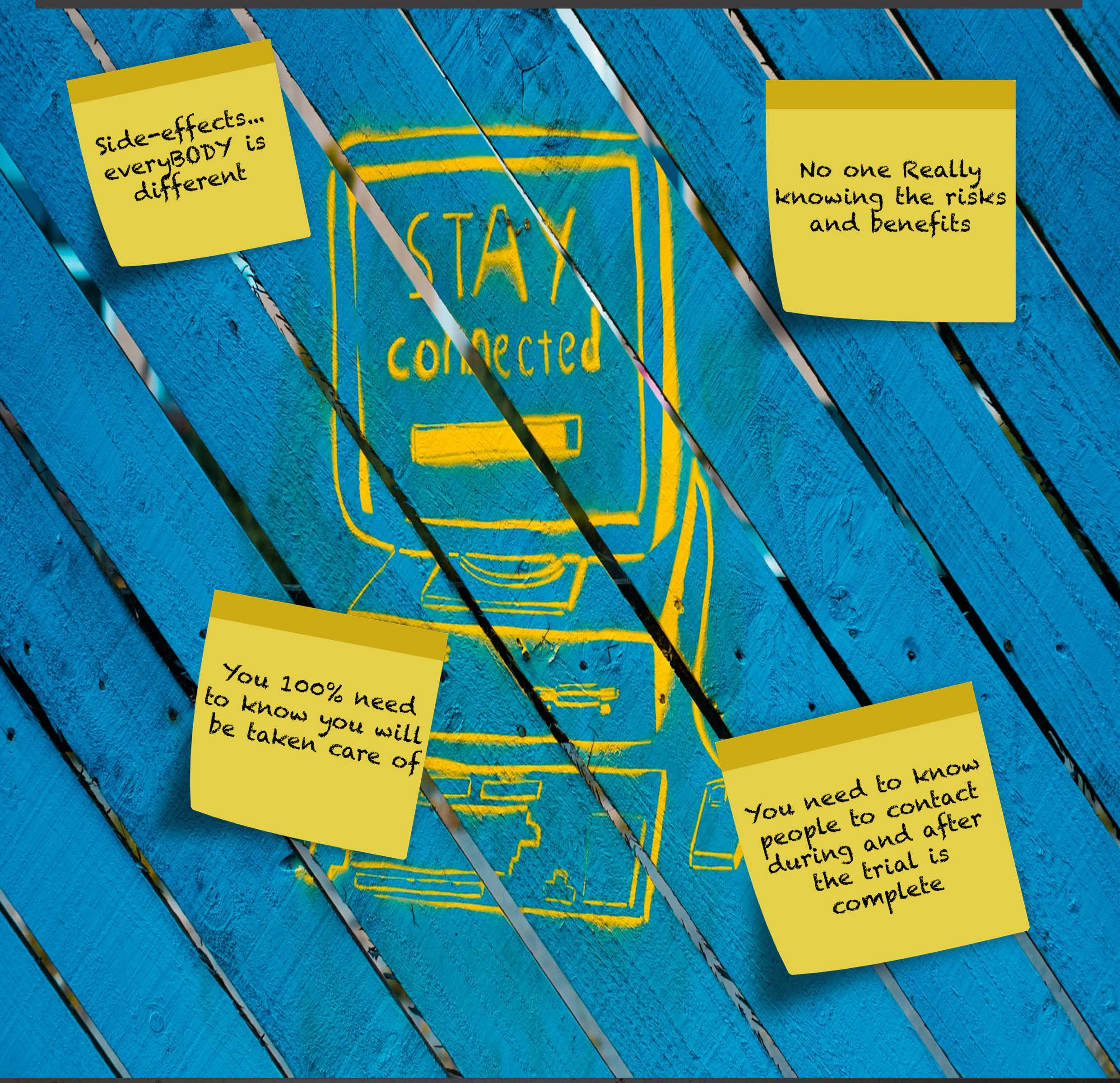

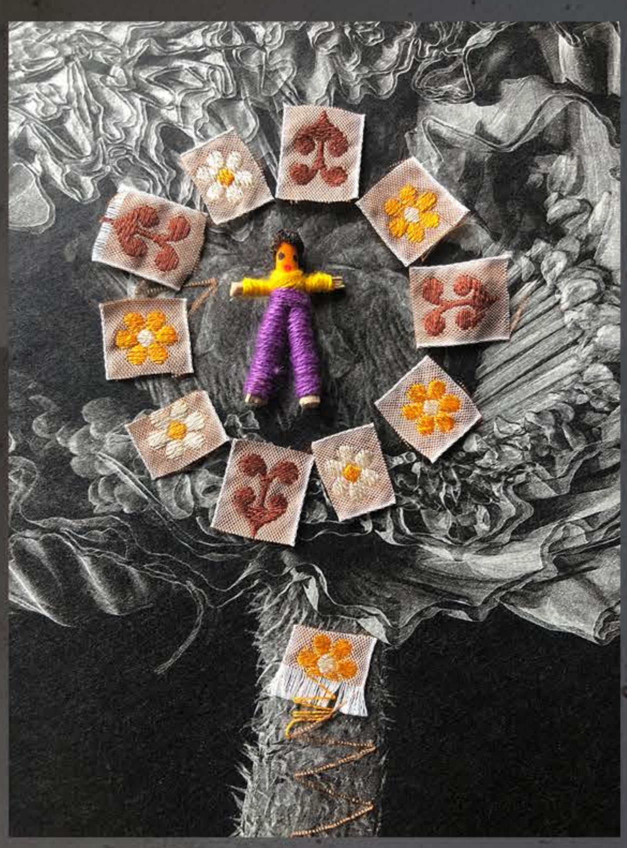

You only hear when things go wrong - we need to tell the good stories

It is exciting to be part of a new initiative which will be beneficial

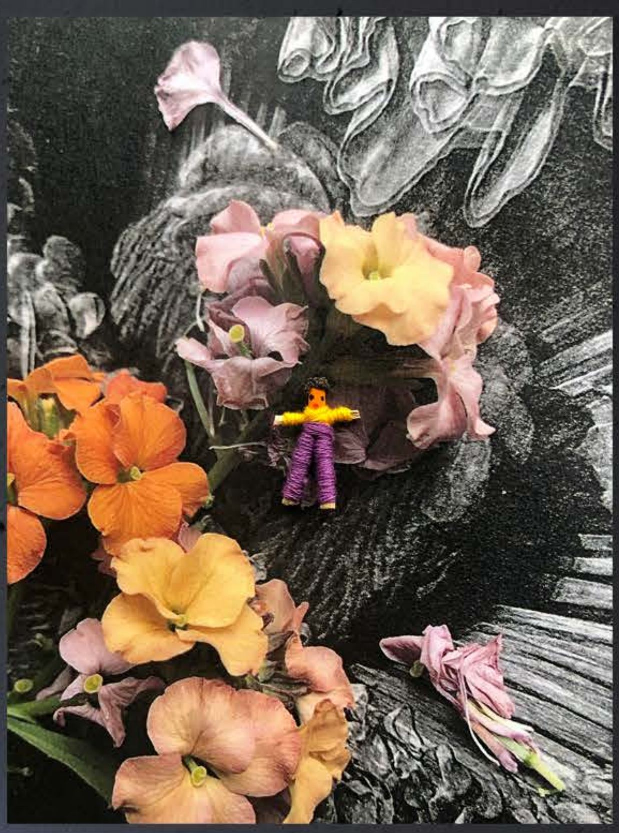

“Talking Trials”

THE HEAD AND THE HEART

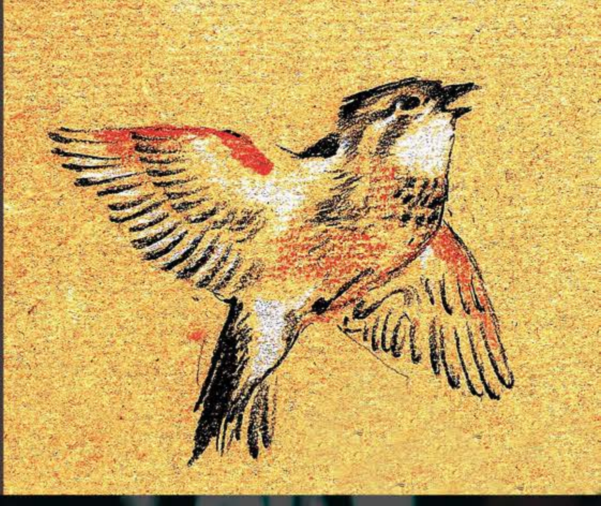

A man in two worlds “Do’s and Don’ts”

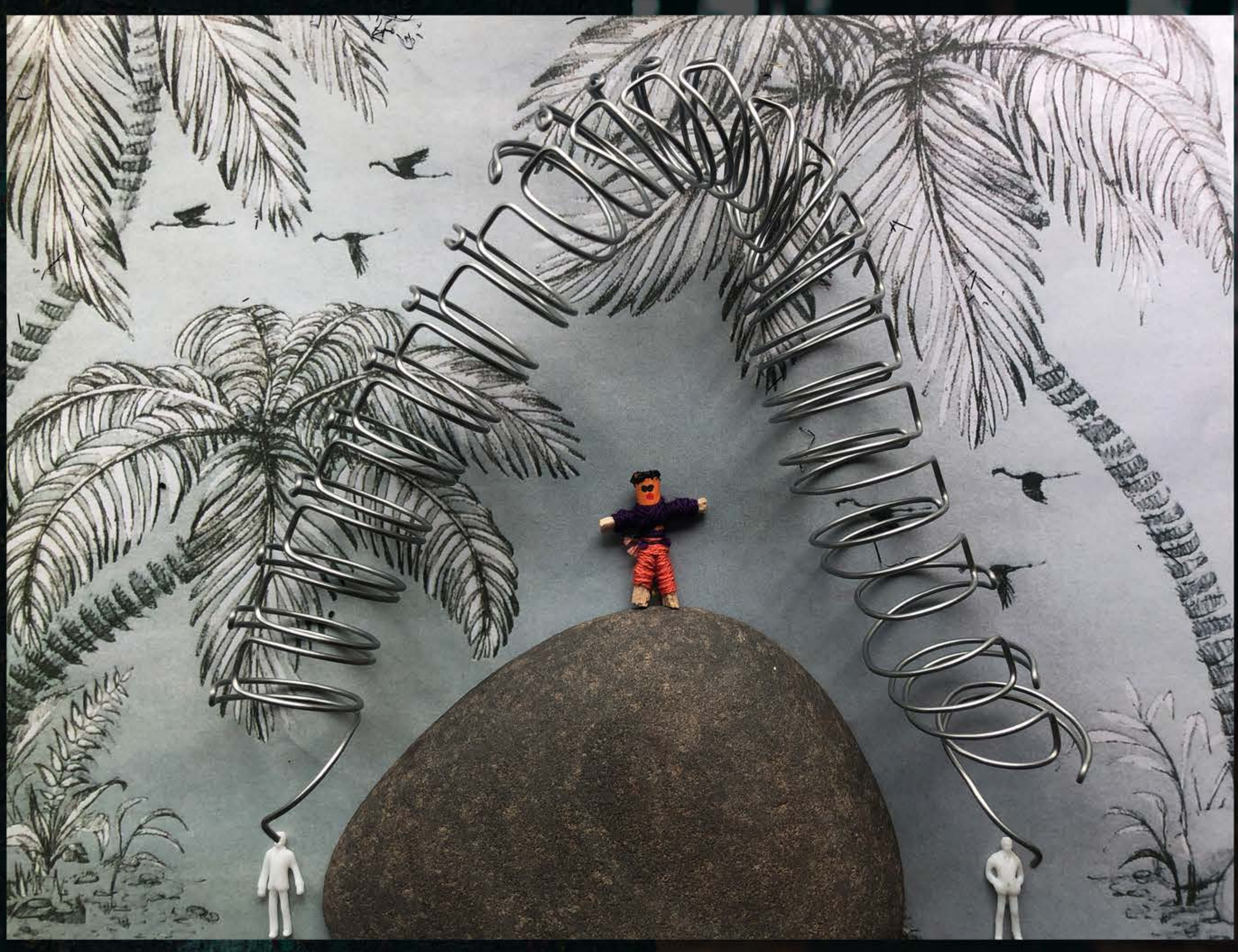

Thinking so hard, on an island

Floating in the Sea of Uncertainty, not knowing which way to go

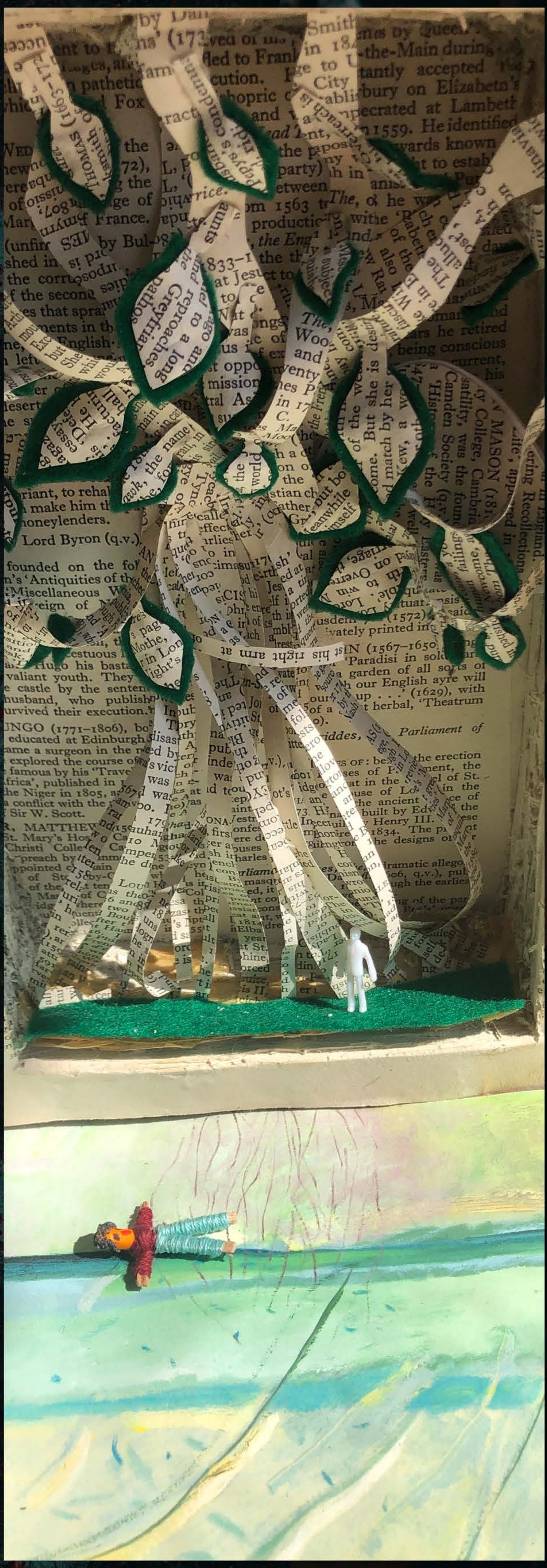

Looking to the man in the white coat and the Tree of Knowledge

This represents my anxiousness about a Clinical Trial, I’m having a sleepless night

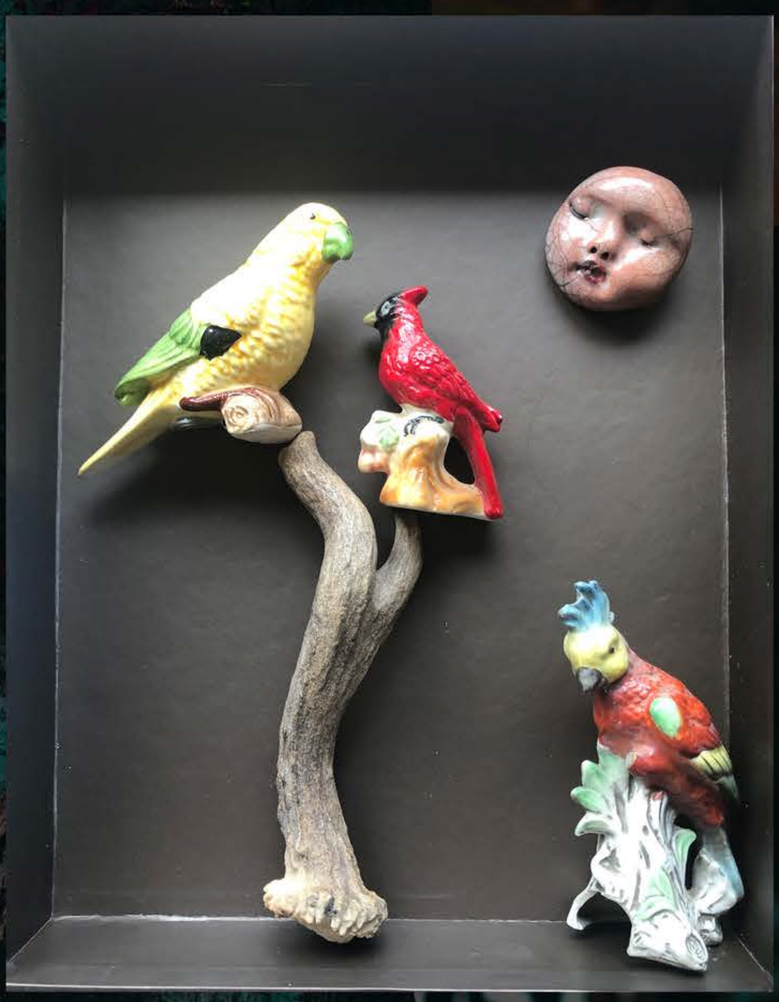

I feel like my mind and body are disconnected, remembering the birdsong in the morning and the sunrise helps me to do this

“Talking Trials”

WE NEED TO TALK

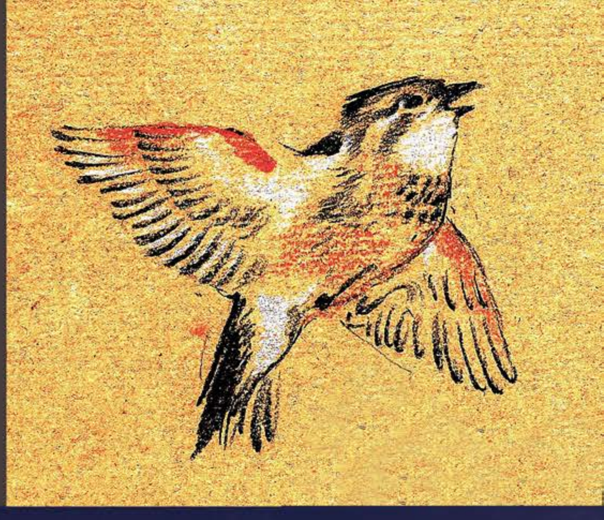

Although we are are all different, our fears, phobias and basic emotions are all the same

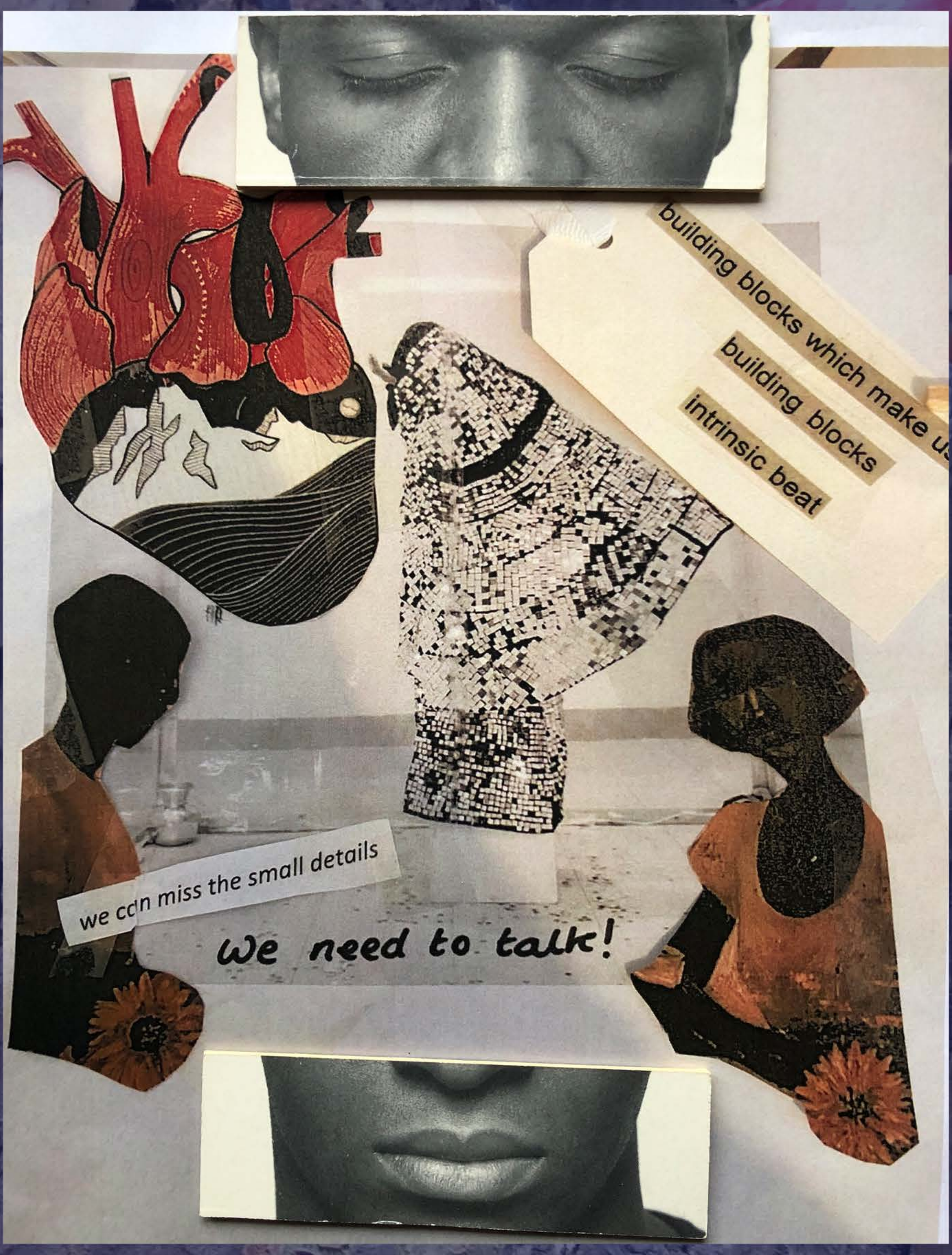

We need to feel safe and confident that our needs are being met

Building trust
Respecting individuality

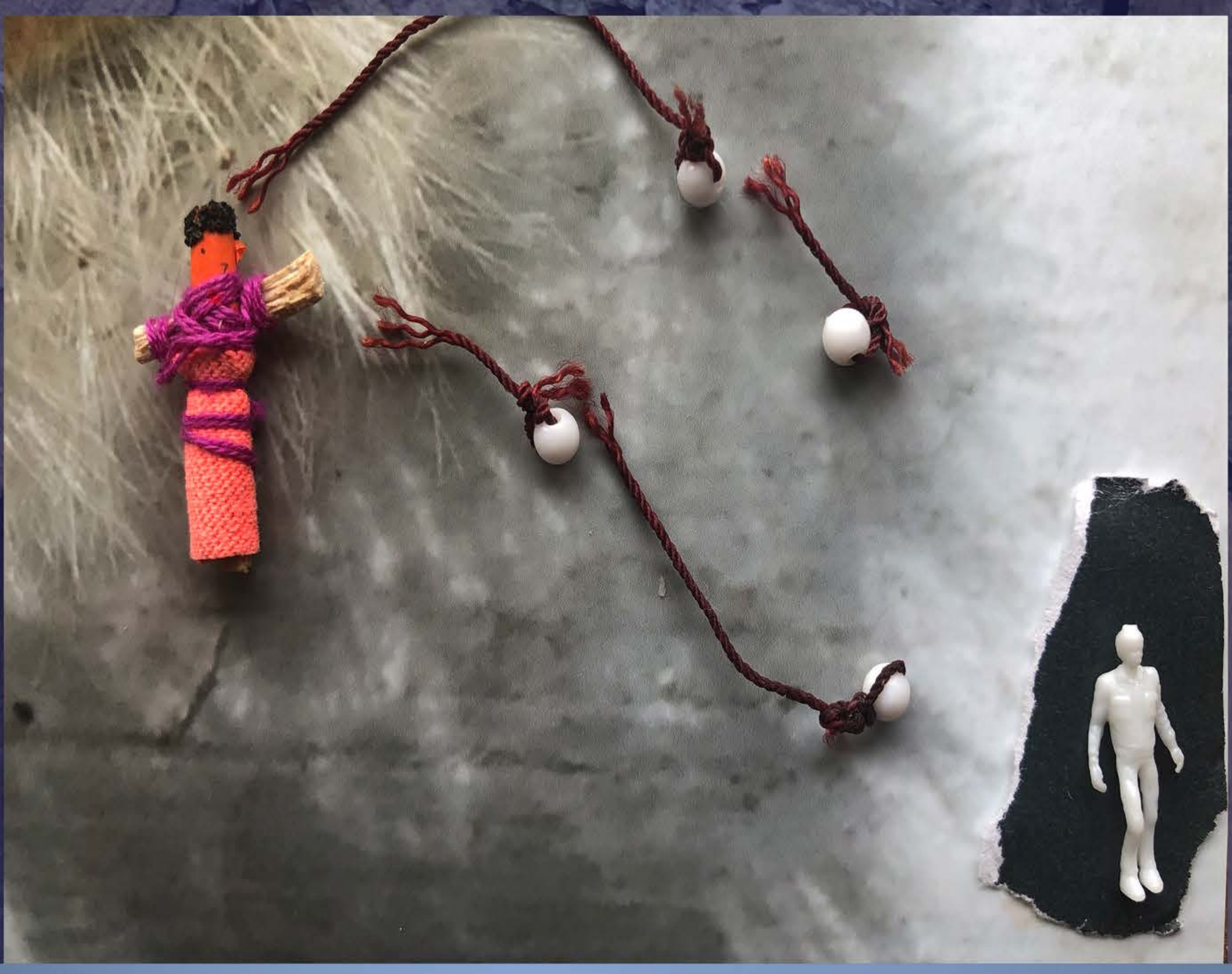

Relying on the doctor to explain and share trial information

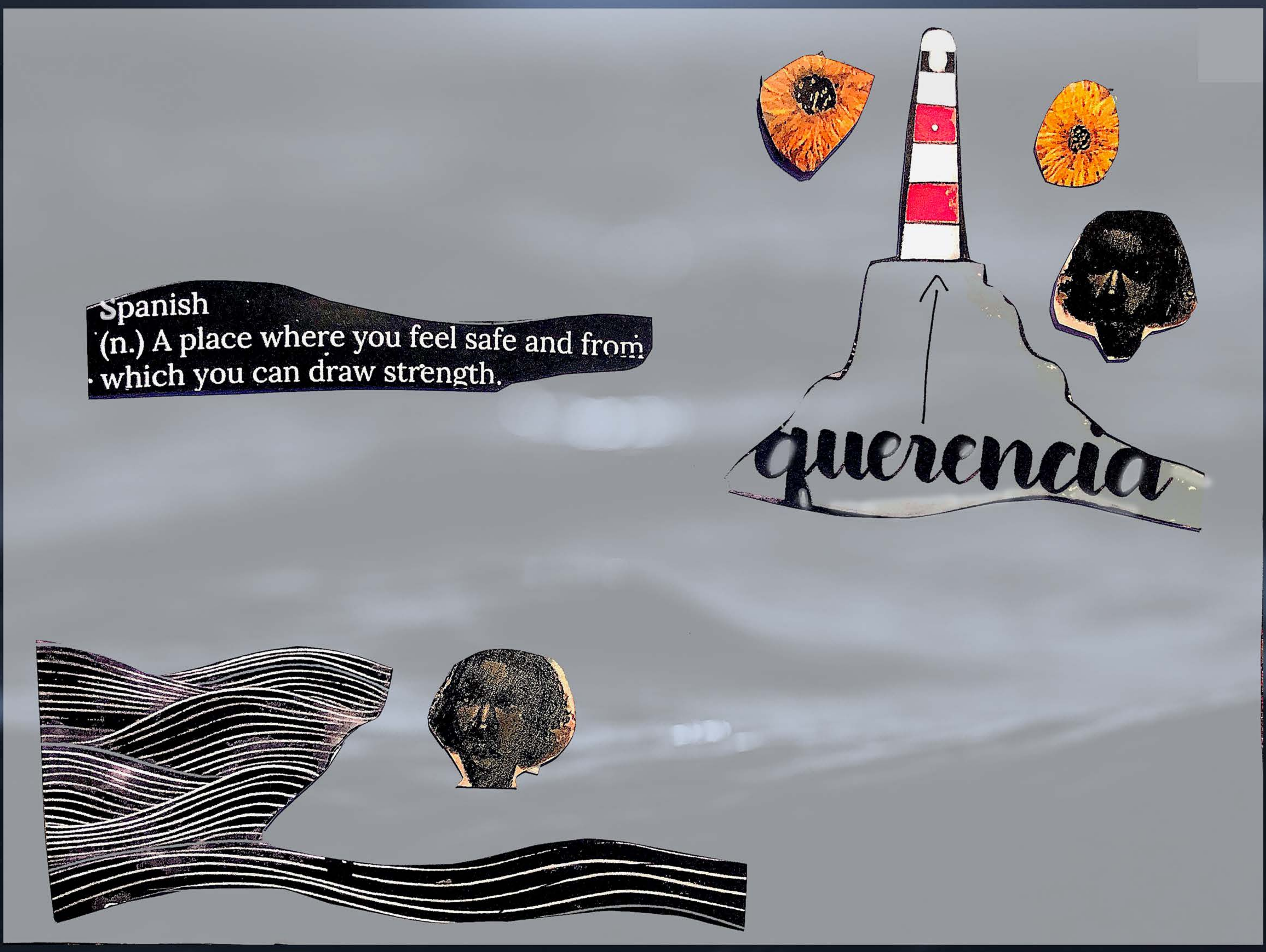

“Talking Trials”

CONNECTED COMMUNITIES AND THE FUTURE

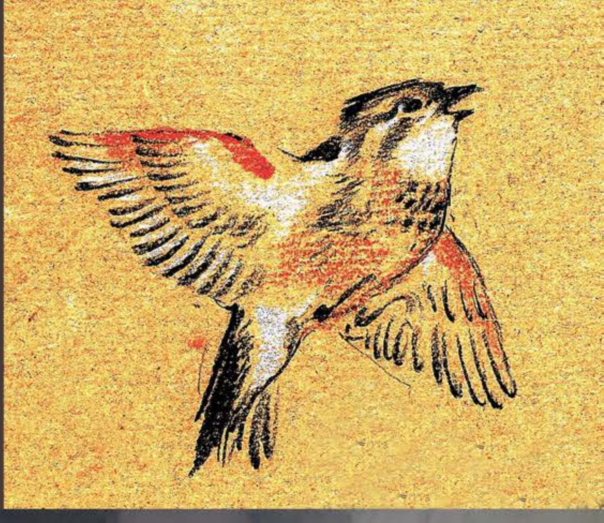

Leaving our children a better future as our roots grow strong

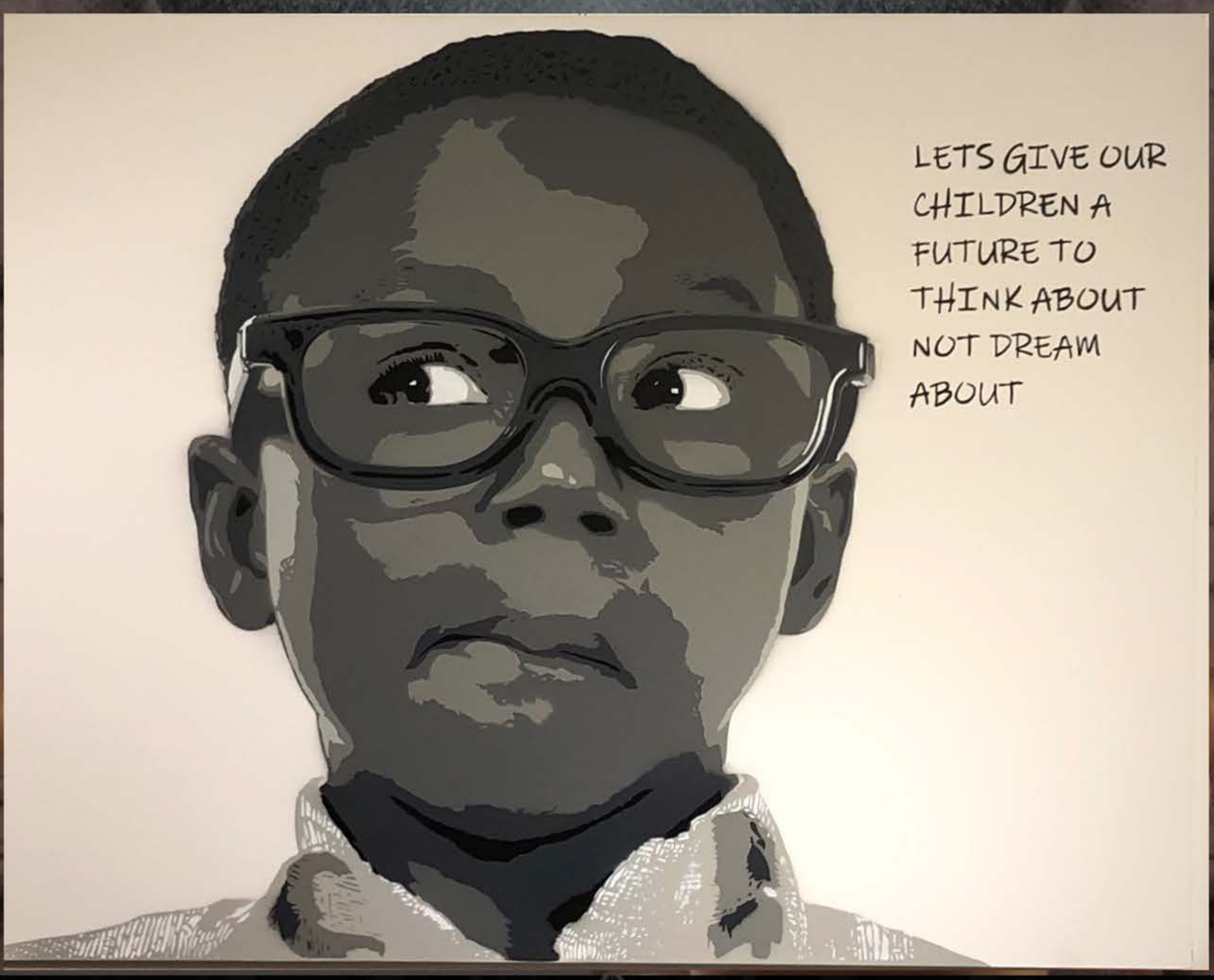

All together, going forward as one

This tree represents the ‘tree of life’

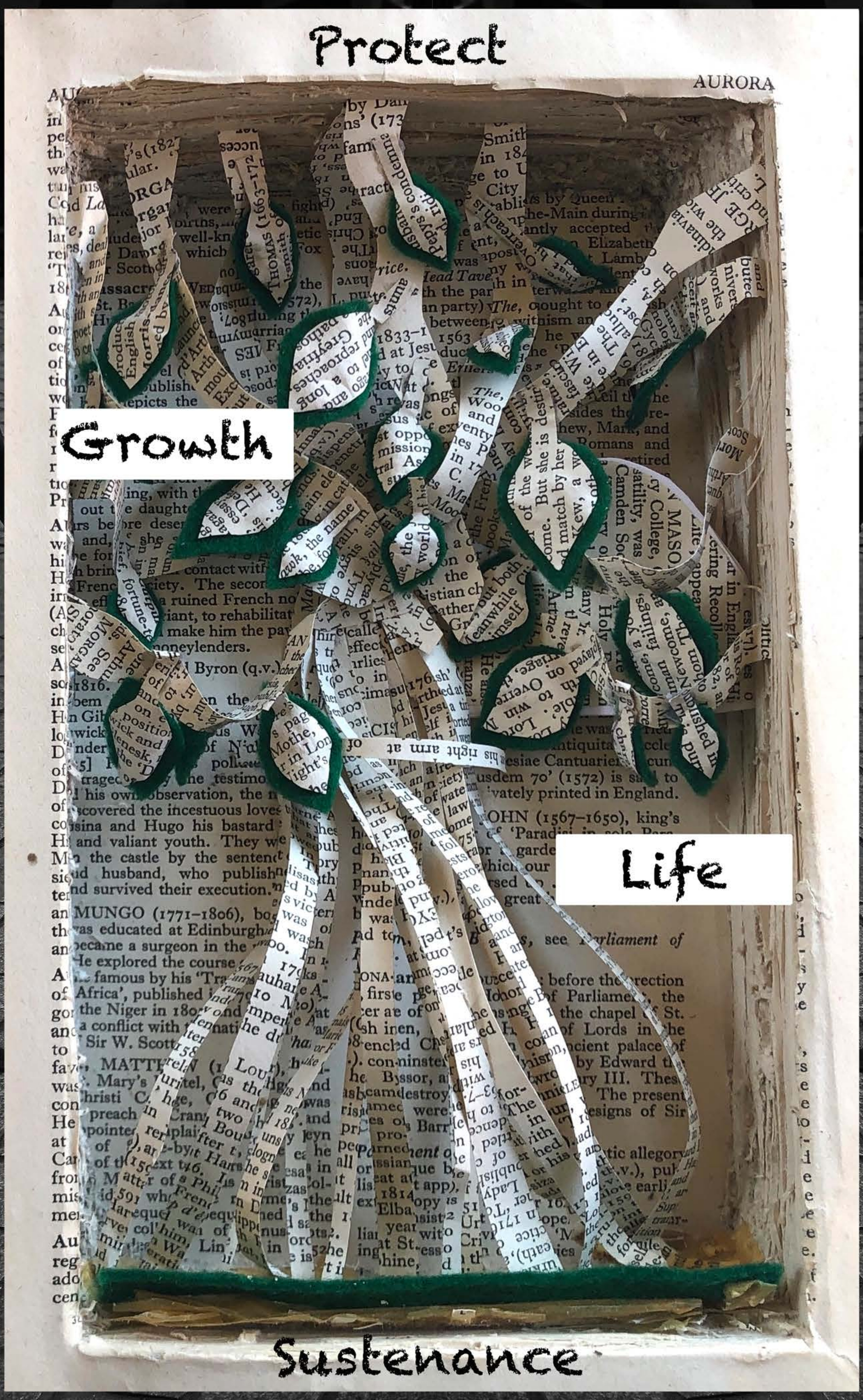

It keeps us grounded and connected

The different branches represent the different people and cultures

Being invited to contribute is appreciated - what YOU bring to the trial counts

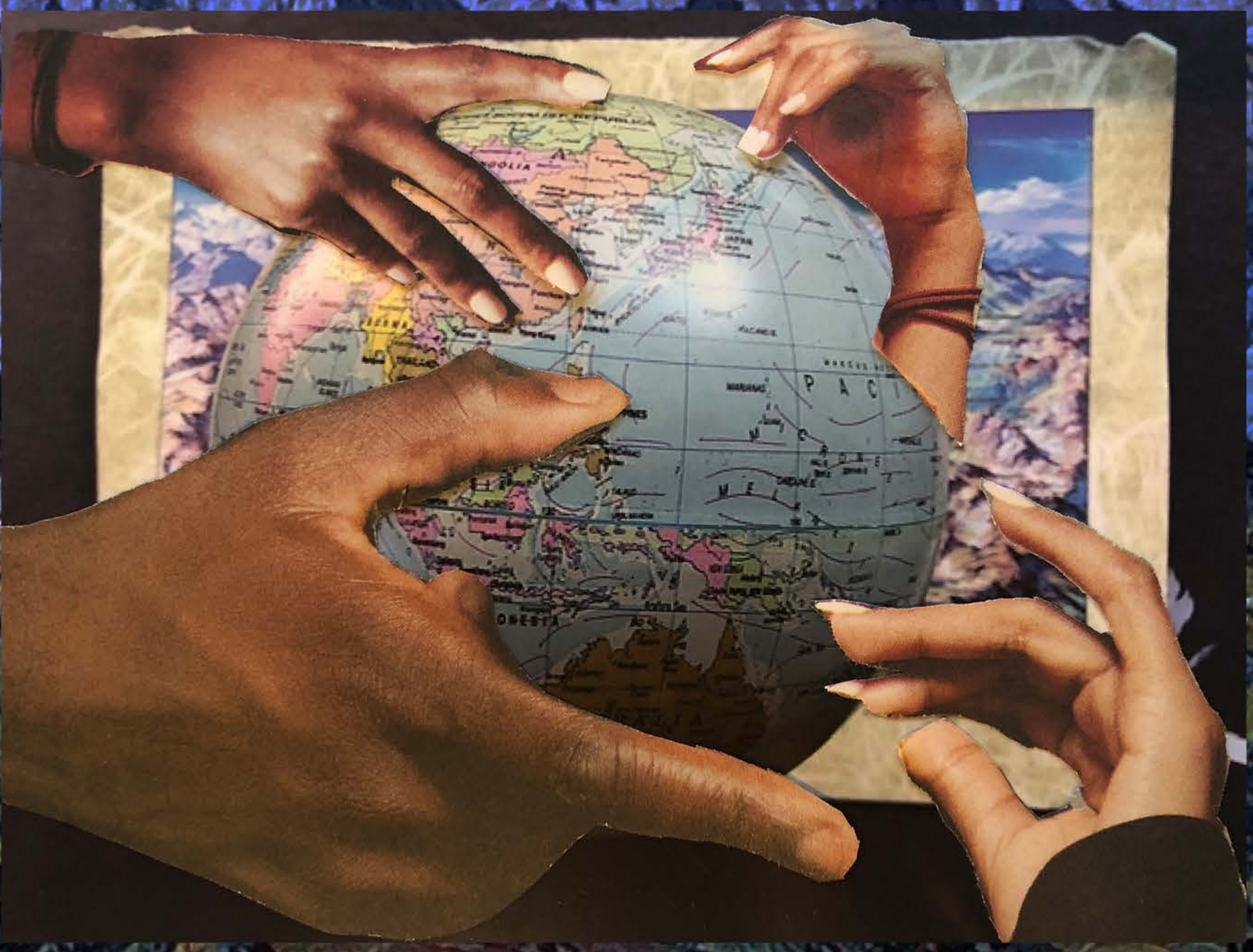

Contributing to a better future because YOUR body is in the mix

A Medical Trial is a Gift - A Helping Hand

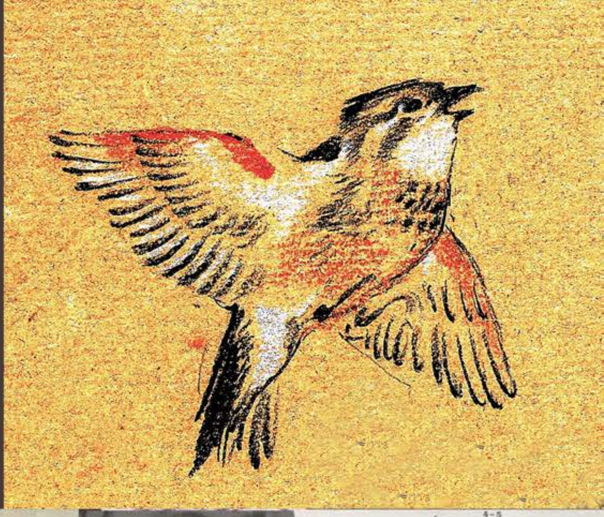

ACKNOWLEDGEMENTS

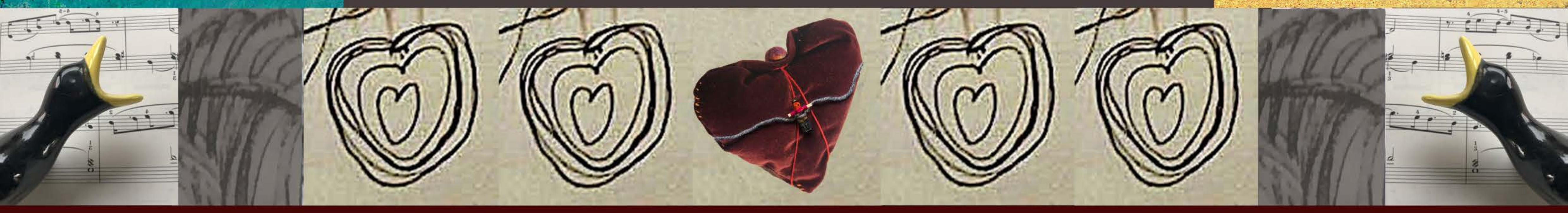

Special thanks and appreciation to all the Talking Trials co-researchers

Arafa Ali
Ayesha Mita
Saleema Bibi
Nasra Ahmed
Mamadu Saliu Djalo
Sudipta Bandyopadhyay
Filomeno Fernandes Gomes Cuino
Sonia Dabo
Mariama Nelida Gomes Correia
Fatima Mussa Abdelmoula Mahmoud
Rossana Canu
Alka Horne
Kense Hayan

We would also like to thank those who helped the project to happen

Allan Herbert
Mashmooma Din
Sarra Ibrahim
Jim Fitzgibbon
Nick Lewis
Carl Smith
Julia Townson
Carl Phillips

South Riverside Community Development Centre.
The River Music Project

Sarah Bridges
Martina Svobodova

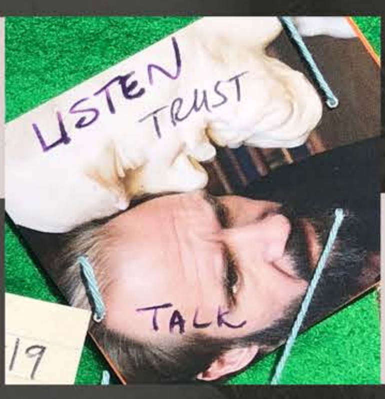

Talking Trials Lead Researchers
Centre for Trials Research

Artists whose works have inspired co-researchers

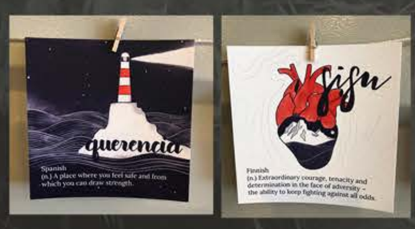

Kiwani Dolean – Fifu and Querencia
https://www.kiwanidolean.com/portfolio/weird-wonderful-words

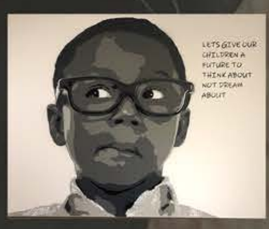

Jon D'oh Bristol Street Artist
Future To Think About
http://www.john-doh.co.uk

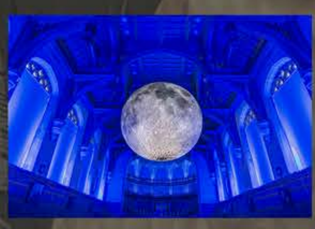

Carolyn Eaton - Dorset Moon
https://www.instagram.com/carolyn.eaton (Luke Jerram Project)

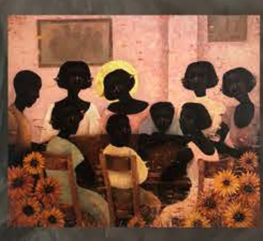

Chidinma Nnoli - Family
http://www.rele.co

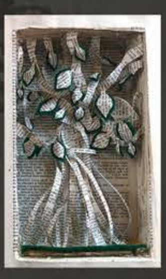

Oshi Weerasinghe – Experience. Inspiration. Knowledge. Growth.
https://outofourheads.net

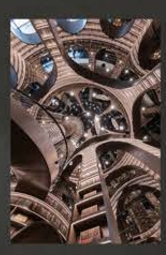

X+living - Dujiangyan Zhongshuge Library Project @xlivingart. Photographer Shao Feng

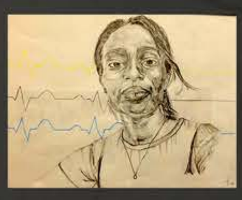

Essi Igwelaezoh - A Portrait in Time
https://outofourheads.net

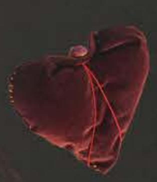

Jan Connett - Heartfelt Exhibition
Bristol Community Arts Project

(Selected photographic backgrounds from https://unsplash.com)

Cardiff University / Prifysgol Caerdydd — Centre for Trials Research / Canolfan Ymchwil Treialon
Ymchwil Iechyd a Gofal Cymru / Health and Care Research Wales
Ariennir gan Lywodraeth Cymru / Funded by Welsh Government
Cancer Research UK
wellcome
South Riverside Community Development Centre

Exhibition curated by
Catherine Lamont-Robinson

SCAN ME
